# The insight into the biology of five homologous lectins produced by the entomopathogenic bacterium and nematode symbiont *Photorhabdus laumondii*

**DOI:** 10.1093/glycob/cwaf033

**Published:** 2025-06-03

**Authors:** Eva Paulenová, Pavel Dobeš, Filip Melicher, Josef Houser, Lukáš Faltinek, Pavel Hyršl, Michaela Wimmerová

**Affiliations:** Central European Institute of Technology, Masaryk University, Kamenice 5, Brno 625 00, Czech Republic; National Centre for Biomolecular Research, Faculty of Science, Masaryk University, Kotlářská 267/2, Brno 611 37, Czech Republic; National Centre for Biomolecular Research, Faculty of Science, Masaryk University, Kotlářská 267/2, Brno 611 37, Czech Republic; Department of Experimental Biology, Faculty of Science, Masaryk University, Kotlářská 267/2, Brno 611 37, Czech Republic; Central European Institute of Technology, Masaryk University, Kamenice 5, Brno 625 00, Czech Republic; National Centre for Biomolecular Research, Faculty of Science, Masaryk University, Kotlářská 267/2, Brno 611 37, Czech Republic; Central European Institute of Technology, Masaryk University, Kamenice 5, Brno 625 00, Czech Republic; National Centre for Biomolecular Research, Faculty of Science, Masaryk University, Kotlářská 267/2, Brno 611 37, Czech Republic; Department of Biochemistry, Faculty of Science, Masaryk University, Kotlářská 267/2, Brno 611 37, Czech Republic; Department of Experimental Biology, Faculty of Science, Masaryk University, Kotlářská 267/2, Brno 611 37, Czech Republic; Central European Institute of Technology, Masaryk University, Kamenice 5, Brno 625 00, Czech Republic; National Centre for Biomolecular Research, Faculty of Science, Masaryk University, Kotlářská 267/2, Brno 611 37, Czech Republic; Department of Biochemistry, Faculty of Science, Masaryk University, Kotlářská 267/2, Brno 611 37, Czech Republic

**Keywords:** glycan array, lectin, nematode, *Photorhabdus*, structure-function study

## Abstract

*Photorhabdus laumondii* is a well-known bacterium with a complex life cycle involving mutualism with nematodes of the genus *Heterorhabditis* and pathogenicity towards insect hosts. It provides an excellent model for studying the diverse roles of lectins, saccharide-binding proteins, in both symbiosis and pathogenicity. This study focuses on the seven-bladed β-propeller lectins of *P. laumondii* (PLLs), examining their biochemical properties (structure and saccharide specificity) and biological functions (gene expression, interactions with the nematode symbiont, and the host immune system response). Structural analyses revealed diverse oligomeric states among PLLs and a unique organisation of binding sites not described outside the PLL lectin family. Lectins exhibited high specificity for fucosylated and *O*-methylated saccharides with a significant avidity effect for multivalent ligands. Gene expression analysis across bacterial growth phases revealed that PLLs are predominantly expressed during the exponential phase. Interaction studies with the host immune system demonstrated that PLL5 uniquely induced melanisation in *Galleria mellonella* hemolymph. Furthermore, PLL2, PLL3, and PLL5 interfered with reactive oxygen species production in human blood cells, indicating their potential role in modulating host immune responses. Biofilm formation assays and binding studies with nematode life stages showed no significant involvement of PLLs in nematode colonization. Our findings highlight the primary role of PLLs in *Photorhabdus* pathogenicity rather than in symbiosis and offer valuable insight into the fascinating dynamics within the *Photorhabdus*-nematode-insect triparted system.

## Introduction

Bacteria from the genus *Photorhabdus* are known for living in symbiosis with entomopathogenic nematodes (EPNs) of the genus *Heterorhabditis*. This nematobacterial complex consisting of highly effective insect pathogens is commercially produced and used as a valuable alternative to chemical pesticides to protect plants against insect pests, such as scarab grubs (*Coleoptera*), sweet potato weevil (*Cylas formicarius*) or corn rootworm (*Diabrotica* spp.) (Lacey et al. 2015). Moreover, *Photorhabdus* spp. is known for its extensive production of secondary metabolites with insecticidal, anti-fungal, and anti-bacterial activity (Cimen et al. 2022). A new antibiotic selective for Gram-negative bacteria, darobactin, was isolated from *Photorhabdus* recently (Imai et al. 2019).

The complex *Heterorhabditis*-*Photorhabdus* is an excellent candidate for studying the molecular mechanisms underpinning pathogenicity and mutualism, as well as the switch between these two life strategies (Clarke 2008). The basics of *Heterorhabditis-Photorhabdus* biology have been studied for decades and are well described (Forst et al. 1997; Waterfield et al. 2009; Clarke, 2020). *Photorhabdus* is carried in an intestine of the specialized larval stadium of *Heterorhabditis*, so-called infective juveniles (IJs). IJs are developmentally arrested and do not feed. They live in the soil and actively look for insect prey. IJs attack the insect larva by natural openings or disrupt the larval cuticle with a tooth-like protuberance (Bedding and Molyneux 1982). Once inside the larval body, IJs release *Photorhabdus* into the hemolymph. *Photorhabdus* produces numerous toxins and other virulence factors and kills the insect host due to septicaemia (Ciche et al. 2008; Waterfield et al. 2009). The bacteria multiply on the insect tissues, and the bacterial mass serves as nutrition, necessary for developing the nematode symbionts (Ciche et al. 2001). In contact with the host, IJs undergo the recovery process and develop through several larval stages in hermaphrodite adults, followed by several sexual reproduction cycles. When the insect tissues are depleted, a new generation of IJs associated with *Photorhabdus* leave the cadaver and look for new prey (Ciche and Ensign 2003; Clarke 2020).

Lectins, in general, are a well-known group of proteins capable of binding saccharide compounds with high specificity (Sharon 2007). They facilitate cell-to-cell interactions on the molecular level in multiple physiological and pathophysiological processes (Sharon 1987; Sharon 2007). They are a perfect tool to read the “glyco-code “present on the surface of every cell. The surface sugar epitopes reflect the type of the cell as well as its current status. By reading this information coded in glycan surface, lectins can mediate, for example, the cell adhesion to the specific tissues or trigger/hinder the immune system reaction in specific locations (Brown et al. 2018).

Bacteria *Photorhabdus* sp. have three distinct and obligatory roles to play in the life cycle of the nematobacterial complex. First, to overcome host defences and kill it; second, to support the nematode growth and development; and third, to colonize the gut of newly developed IJs (Clarke 2020). In all these roles, lectins could mediate interactions through their carbohydrate-binding activity. Several structurally related lectins forming a group with a conserved seven-bladed β-propeller fold (PLL family) were identified in different *Photorhabdus* species: PLL in *P. kayaii* (Kumar et al. 2016), PLL2 and PLL3 in *P. laumondii* (Faltinek et al. 2019; Fujdiarová et al. 2021) and PHL in *Photorhabdus asymbiotica* (Jančaříková et al. 2017). Basic characterization of these lectins revealed a common preference for l-fucose and its derivatives and *O*-methylated saccharides; also the inhibition of reactive oxygen species was described for PLL2 and PHL (Jančaříková et al. 2017; Fujdiarová et al. 2021). Besides PLL family, a structurally unrelated lectin PllA was also identified in *P. laumondii*. PllA specifically recognizes d-galactose, and its function in the *Photorhabdus* life cycle remains unclear (Beshr et al. 2017).

This article brings a wide-ranged comprehensive study of five members of the PLL family originating from a single *Photorhabdus* strain: *P. laumondii* subsp. *laumondii* TT01. Interestingly, genes coding these five lectins are co-localized in the bacterial genome. In this study, we broadly extend the previous research of two of these lectins and compare the results with the newly characterized members. The properties of carbohydrate interactions, atomic structure, and interaction with both insect and nematode hosts are presented and compared. The involvement of PLLs in either mutualism or pathogenicity of *P. laumondii* is investigated in depth.

## Results and discussion

### Lectins involved in the study

In this article, we studied five members of the PLL lectin family from *P. laumondii* subsp. *laumondii* TT01 (PLL, PLL2, PLL3, PLL4, PLL5, collectively referred to as PLLs). All five genes are co-localized in the bacterial genome within a 7.7 kbp region (Fig. S1). Previous studies on PLL were conducted using the native protein isolated from *Photorhabdus kayaii* (formerly *Photorhabdus luminescens* subsp. *kayaii*) and its recombinant version containing a His-tag for protein purification (Kumar et al. 2016). In this study, we used PLL originating directly from *P. laumondii* subsp. *laumondii* TT01 (formerly *P. luminescens* subsp. *laumondii* TT01) to ensure all five studied lectins originate from the same bacterial strain. The two versions of PLL differ in 12 mutated amino acids – five of them affecting three potential binding sites. Additionally, the protein used in this study lacks the His-tag on the C-terminus and is seven residues longer on the N-terminus (Fig. S2). The observed differences in PLL behaviour between this study and earlier reports justify our approach and highlight the importance of not neglecting minor changes in the studied protein constructs.

The production of PLL2 and PLL3 has been already described (Faltinek et al. 2019; Fujdiarová et al. 2021). The newly characterized lectins PLL, PLL4, and PLL5 were produced recombinantly in *Escherichia coli* and were purified by affinity chromatography. All proteins form a single band on the SDS-PAGE gel with an apparent molecular mass of ~40 kDa, and their identity was verified using MALDI MS/MS and intact mass analyses (Figs. S3, S4, and  S5).

### Expression of genes encoding PLLs during bacterial growth

**Fig. 1:**
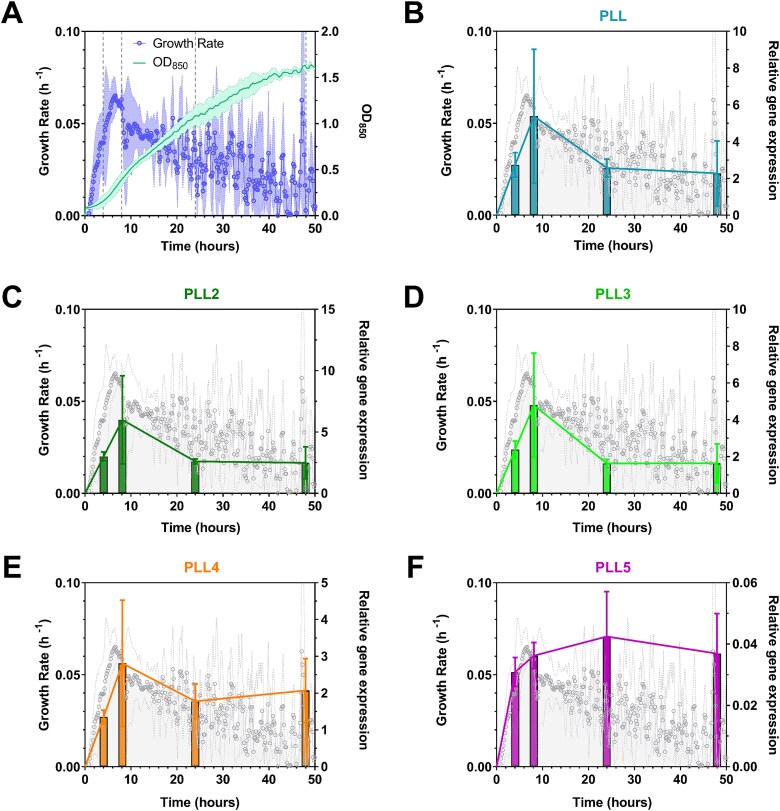
Growth characteristics of *Photorhabdus laumondii* and relative expression of genes coding for PLLs. All data represent the mean ± SD of three independent experiments. A) the growth of *P. Laumondii* in liquid medium was monitored every 10 min over two days with regular measurements of optical density at 850 nm (OD_850_) and growth rate. Vertical dashed lines indicate the time points at which samples for gene expression analysis were collected. B-F) the relative expression of genes encoding PLLs was measured at time points 4, 8, 24, and 48 h after inoculation. Statistical analysis revealed that changes in gene expression correlated with bacterial growth (Kruskal–Wallis test with post hoc Dunn’s multiple comparisons test; *P* < 0.05).

There is a clear correlation between *Photorhabdus* pathogenicity and the bacterial exponential growth phase, while the late stationary phase resembles a stable *Photorhabdus* population that supports the growth of symbiotic nematodes (Ffrench-Constant et al. 2003). To investigate the biological potential of PLLs, we measured their gene expression in *P. laumondii* TT01 grown in a liquid medium. This approach was reported to be comparable with *Photorhabdus* grown within the infected host (Daborn et al. 2001). The selected time points at 4, 8, 24, and 48 h post-inoculation corresponds to lag, exponential, late exponential, and early stationary growth phases, respectively (Fig. 1A).

Our findings indicate that lectins from the PLL family, except for PLL5, are produced by *P. laumondii* TT01 simultaneously at all time points (Fig. 1B-F). The gene expression parallels the growth phases of the bacterium. The highest expression levels were observed during the exponential growth phase, indicating the role of PLLs in *Photorhabdus* pathogenicity over mutualism. However, since production was observed at all time points in our experiments, we cannot definitively exclude the possible involvement of PLLs in symbiosis based solely on this data.

The PLL5 gene exhibited negligible expression under the conditions tested, raising questions about its inducibility. This might suggest that *pll5* expression requires some additional external trigger, which could be further explored through in vivo analysis of *Photorbadus*-infected hosts.

### Interaction with innate immune systems

The interaction of PLLs with the host immune system was tested by measuring the phenoloxidase (PO) activity and by monitoring the production of reactive oxygen species (ROS). Both mechanisms have been previously described in both invertebrates and vertebrates as being modulated during pathogen recognition and initial defense response against it (Kumar et al. 2003; Cerenius and Söderhäll 2004).

**Fig. 2:**
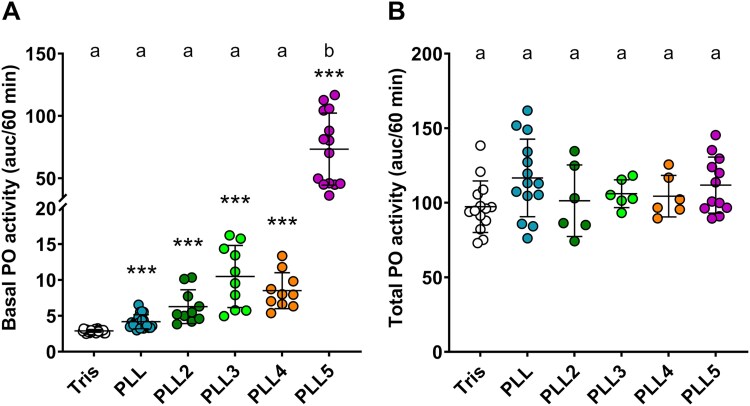
Basal activity of phenoloxidase (PO) and total phenoloxidase activity in the hemolymph of *G. Mellonella* incubated with PLLs. A) the melanisation of hemolymph treated with PLLs was quantified by measuring the absorbance at 492 nm for 60 min. The results are expressed as the area under the curve. B) To measure total PO activity, hemolymph samples were pre-treated with α-chymotrypsin. Data are presented as the mean ± SD; *n* ≥ 6. Different letters above columns indicate significant differences *P* < 0.05 among all treatments (Tukey’s test); asterisks represent a significant difference in pairwise comparison between Tris control and individual proteins (^***^P < 0.001).

Basal PO activity was measured in cell-free hemolymph collected from *Galleria mellonella* larvae. We found that all PLLs can trigger a melanisation response compared with the buffer control (Fig. 2A; t-tests, *P*-values <0.001 for each comparison), so they are likely recognised as pathogen-associated molecules by the host immune system. This finding confirmed our previous results with PLL2 and the homologous PHL lectin (Fujdiarová et al. 2021). Among the PLL family, PLL5 had the most pronounced effect on basal PO activity (one-way ANOVA, *P*-value <0.001), elevating it to levels comparable to those seen with full activation of the PO system. These results suggest that PLL5 might play an active role in eliciting melanisation beyond mere recognition by the immune system, similar to other proteins of *Photorhabdus* bacteria (Held et al. 2007; Li et al. 2014). Overactivation of the PO system might help establish a successful infection by depleting the prophenoloxidase reserves, exhausting and confusing the insect’s innate immune response in a temporally or spatially controlled manner. Nonetheless, the extent of PLL5-induced melanisation under natural infection is uncertain due to low *pll5* gene expression in early bacterial growth stages. None of the tested proteins affected the total PO activity measured after the proteolytic activation of prophenoloxidase (Fig. 2B), indicating that their positive effects on basal PO activity are likely to be non-genomic.

**Fig. 3:**
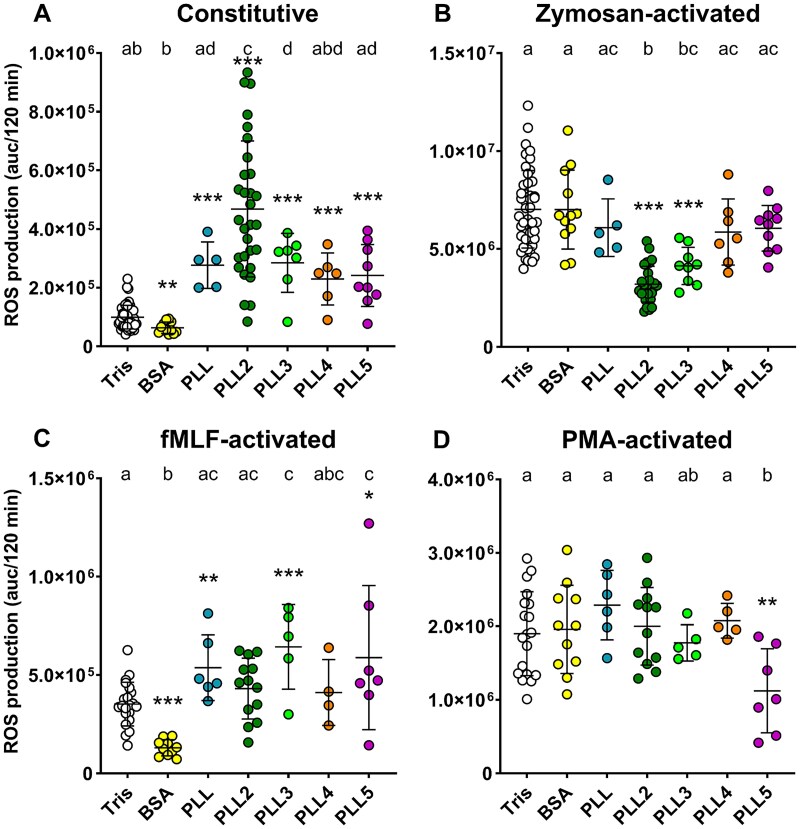
Production of reactive oxygen species (ROS) in human blood treated with PLLs. (A) the constitutive ROS production in human blood treated with PLLs was measured luminometrically for 120 min at 37 °C and expressed as an area under the curve. To determine effect of PLLs on activated neutrophils, the reaction was also measured in the presence of activators: Zymosan a (B), fMLF (C), and PMA (D). Data are presented as the mean ± SD; *n* ≥ 4. Different letters above columns indicate significant differences *P* < 0.05 among all treatments (Tukey’s test); asterisks represent a significant difference in pairwise comparison between Tris and individual protein (^*^P < 0.05; ^**^P < 0.01; ^***^P < 0.001).

Our previous research demonstrated that PLL2 and PHL lectins produced by *Photorhabdus* spp. interact with human blood cells, leading to the increased ROS production (Jančaříková et al. 2017; Fujdiarová et al. 2021). In this study, we confirmed that PLL2 highly increased ROS production and discovered that other PLLs also activate a similar oxidative response (Fig. 3A), suggesting that the immune systems of both humans and insects recognise all these lectins.

To simulate the effect of PLLs on the innate immune system reaction during the ongoing infection, the ROS production was induced by one of the potent activators – zymosan A, formyl-methionyl-leucyl-phenylalanine (fMLF) or phorbol 12-myristate 13-acetate (PMA). The effect of individual PLLs on such an activated system was evaluated. In human blood activated by zymosan A, PLL2 and PLL3 inhibited ROS production, suggesting these lectins interfere with the innate immune system reaction in the early stages of the *Photorhabdus* infection. Other PLLs did not interfere with the zymosan-induced response (Fig. 3B). On the other hand, the oxidative response induced by fMLF was enhanced by PLL, PLL3 and PLL5 (Fig. 3C). This additive effect implies that PLL, PLL3 and PLL5 may induce ROS production independently of fMLF receptors. Interestingly, PLL5 was observed to reduce ROS production in PMA-induced response (Fig. 3D), although a significant variability was noted among blood samples, likely reflecting inter-individual differences among blood donors. PMA activates protein kinase C in the intracellular signalling pathway. The mechanism by which PLL5 inhibits PMA-induced ROS production remains unknown, but it highlights PLL5’s unique activity among other PLLs.

The interference of several PLL lectins with the PO activation and ROS production supports the hypothesis that *Photorhabdus* uses these lectin*s* during the early stages of infection to help overcome the host immune system reaction.

### Interaction with the nematode symbionts

Some of the mechanisms involved in establishing the *Photorhabdus*-*Heterorhabditis* mutualistic relationship are well reviewed in (Clarke 2014); however, the entire process remains not fully understood, and the role of lectins in mediating nematode-bacteria interaction is understudied. Given the reduced number of lectin-encoding genes in *Heterohabditis bacteriophora* (Clarke 2014) and the binding of *Photorhabdus* to nematode cells during the colonisation of infective juveniles (Ciche et al. 2008; Somvanshi et al. 2010), *Photorhabdus* lectins could play a significant role in symbiosis. Therefore, we evaluated the potential of PLLs to mediate the interaction between nematodes and *Photorhabdus* bacteria.

**Fig. 4:**
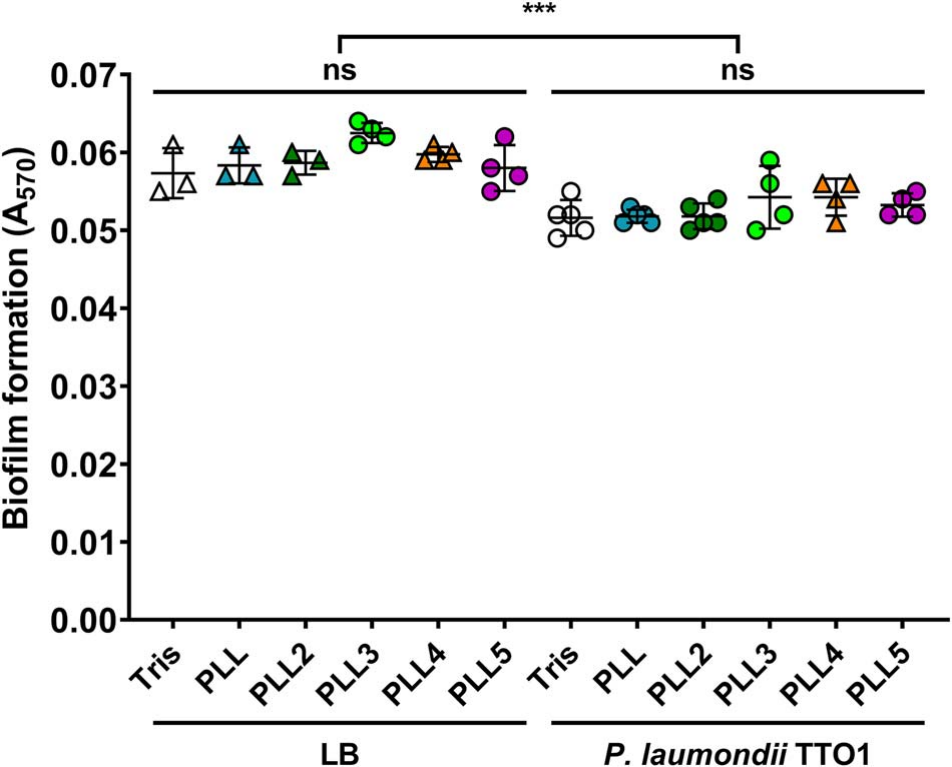
The effect of PLLs on biofilm formed by *P. Laumondii* TT01. PLLs were co-incubated with either the P. Laumondii TT01 suspension or sterile LB medium. The potentially produced biofilm was measured as the absorbance at 570 nm released from crystal violet staining. Data are presented as the mean ± SD; each symbol in the plot represents an individual measurement. No significant differences in biofilm formation were observed among PLLs and Tris buffer (Tukey’s test; *P* > 0.05). The statistically significant difference between the co-incubation of *P. Laumondii* with PLLs and sterile LB medium with PLLs (unpaired t test; *P* < 0.05) suggests non-specific binding of the dye to the cultivation plastic.

We tested biofilm formation, a process supporting *Photorhabdus*-*Heterorhabditis* symbiosis, by co-incubating bacterial culture or sterile LB medium with PLLs. We did not observe any effect of PLLs on the biofilm layer produced by *P. laumondii* TT01 in liquid culture (Fig. 4), indicating they do not participate in nematode colonisation. The incubation of PLLs in LB without bacteria led to a significantly higher adherence of the staining solution to the cultivation plates, suggesting a non-specific binding. Our results confirm previously observed low biofilm formation by *P. laumondii* subsp. *laumondii* TT01 (Zamora-Lagos et al. 2018).

**Fig. 5:**
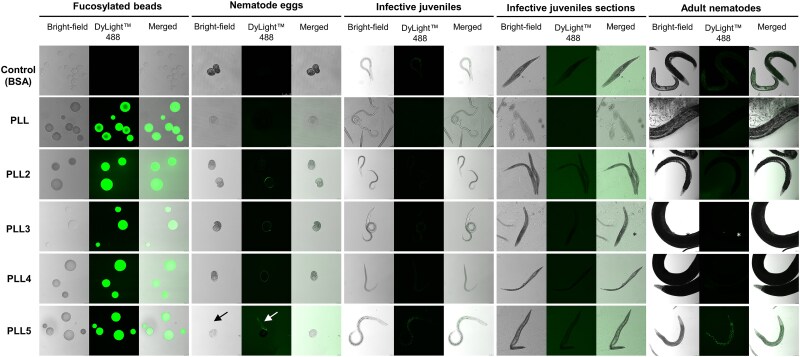
The binding of DyLight™ 488-labelled PLLs to developmental stages of nematodes and fucosylated beads (positive control). Labelled lectins were co-incubated with nematodes or beads and observed using confocal microscopy. The arrow shows a nematode egg losing its surface layer with the visible binding of labelled PLL5. Autofluorescence of the vulva in adult female is marked with an asterisk.

Furthermore, we tested the binding of fluorescently labelled PLLs to different life stages of nematode *H. bacteriophora* H221, including the infective juveniles, eggs, and adults (Fig. 5). PLLs did not bind to nematode stages naturally associated with *Photorhabdus* cells, such as infective juveniles and adults. PLLs did not target the cuticle of all larval stages and adults and did not bind to tissues of IJs. However, the binding of labelled lectins was occasionally observed in the digestive tract of hermaphrodites, where they can participate in nematode development through endotokia matricida (Ciche et al. 2008), or in the intestines of larval instars L1 to L4 (data not shown). Since all these nematode stages are feeding, and the experiments were performed with live nematodes, we cannot distinguish between specific binding and simple digestion of labelled PLLs.

Interestingly, we observed weak binding of PLLs to the surface of nematode eggs. Glycosylation profiles can be specific to nematode developmental stages, as in the case of *C. elegans* (Sheikh et al. 2019), *Brugia malayi* (Mersha et al. 2023) or *Oesophagostomum dentatum* (Jiménez-Castells et al. 2017); unfortunately, the detailed glycosylation profile of *Heterorhabditis* eggs is unknown. However the eggs of parasitic nematode *Schistosoma mansoni* contain saccharides with multi fucosylated termini, which are not observed in other developmental stages of this nematode (Hokke et al. 2007). Chitin is an essential component of eggshells in parasitic nematodes (Wharton and Jenkins 1978), and in our previous work, PLL from *P. kayaii* was observed to recognise chitin oligosaccharides (Kumar et al. 2016). Increased expression of the *pll* gene was also detected after exposing *Photorhabdus* secondary cells to plant root exudate (Regaiolo et al. 2020). The most significant expression increase was reported for chitinase, leading to the discovery of chitin degradation by *Photorhabdus*, suggested as a mechanism to protect plant roots against fungal pathogens (Dominelli et al. 2022). The potential chitin-binding ability of PLLs and resulting interactions could play an essential role in the survival of *Photorhabdus* secondary cells in the soil, presenting a promising area for future research.

### Carbohydrate specificity of PLLs

**Fig. 6:**
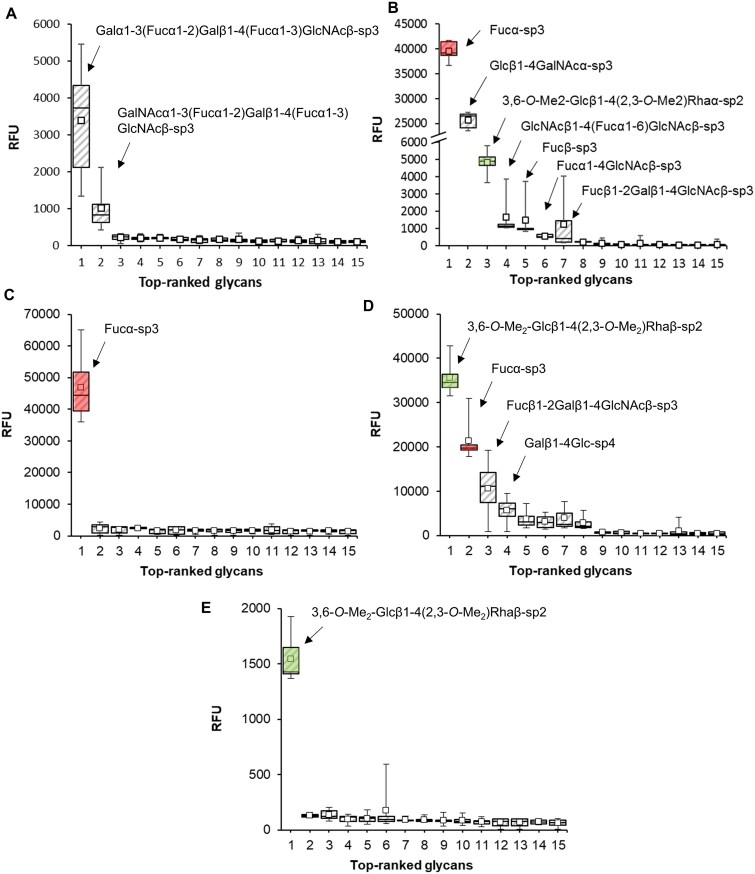
Box and whiskers plot for glycan array screening**.** Lectins (A) PLL, (B) PLL2, (C) PLL3, (D) PLL4 and (E) PLL5 labelled with DyLight488. The top 15 ligands are presented. The saccharides that gave signals with a signal-to-noise ratio > 3 are labelled. Linker formulas are as follows: sp2, -O-(*p*-C_6_H_4_)-O-CH_2_CH_2_NH_2_; sp3, -O-(CH_2_)_3_NH_2_; sp4, -NHCOCH_2_NH_2_. The bottom and top of the box are the first and third quartiles; the band inside the box is the median; the ends of the whiskers represent the minimum and the maximum values of the data, and the small squares inside the boxes represent the mean. RFU, relative fluorescence units. Ligands recognized by at least three lectins are color-coded. Complete glycan array results and raw data are given in supporting Table S4. Data for PLL2 and PLL3 are taken from (Faltinek et al. 2019; Fujdiarová et al. 2021).

To identify lectins’s natural binding partners, a screening of 634 sugar epitopes was performed using a glycan array microchip (Fig. 6). Out of hundreds of possible ligands, the lectins bound to only a few of them, indicating high ligand specificity. Two common ligands were identified: α-l-fucoside (or fucosylated saccharides) and an unusual *O*-methylated disaccharide 3,6-*O*-Me_2_-d-Glcβ1-4(2,3-*O*-Me_2_)-l-Rhaα (MGMR). PLL preferred branched fucosylated saccharides (BLeY and ALeY); PLL4 and PLL5 preferred MGMR. PLL4 also recognized linear d-fucosylated trisaccharide (d-Fucβ1-2-d-Galβ1-4-d-GlcNAcβ), α-l-fucose, and lactose. These results are well in agreement with the binding preferences of other members of the PLL lectin family (PLL2 (Fujdiarová et al. 2021), PLL3 (Faltinek et al. 2019), and PHL (Jančaříková et al. 2017)).

Fucose-containing glycans were described in insects and nematodes (Walski et al. 2017; Jankowska et al. 2018), and fucosylated proteins play a role in the insect immune system (Walski et al. 2017), supporting the potential interaction of PLLs with the insect immune system. MGMR is present in the *Mycobacterium leprae* glycolipid PGL-1 (Hunter et al. 1982; Zhang et al. 2010). *O*-methylation is an unusual sugar modification, described in nematodes and bacteria but not in insects (Staudacher 2012), and is considered a pathogen-associated molecular pattern. It can be recognized by tectonins, a group of β-propeller lectins; e.g. Lb-Tec2 from a mushroom *Laccaria bicolor* the function of which is proposed to be pathogen recognition (Wohlschlager et al. 2014; Sommer et al. 2018). PLLs originating in the pathogenic bacteria are unlikely to have the same function, so the reason for PLLs recognizing *O*-methylation remains unclear. We believe none of the ligands identified on the glycan array are the native ligands of PLLs; however, these sugars could represent the building blocks of the native binding partner.

Detailed analysis of lectins’ interaction with several sugars selected according to glycan-array results was performed using isothermal titration calorimetry (ITC) and surface plasmon resonance (SPR). All dissociation constants determined by ITC are in the low millimolar range (Table S1). Such a low affinity towards monosaccharides is typical for many lectins (Lis and Sharon 1998). The highest affinity was seen towards methyl-α-l-fucoside (αMeFuc), ranging from 0.4 ± 0.02 mM for PLL2 to 4.9 ± 0.08 mM for PLL4. l-fucose was also significantly recognized. PLL2 and PLL3 also interacted with d-galactose, d-glucose, and 3-*O*-methyl-d-glucose (3OMG), while other PLLs did not bind these sugars significantly (Fig. S6).

**Fig. 7:**
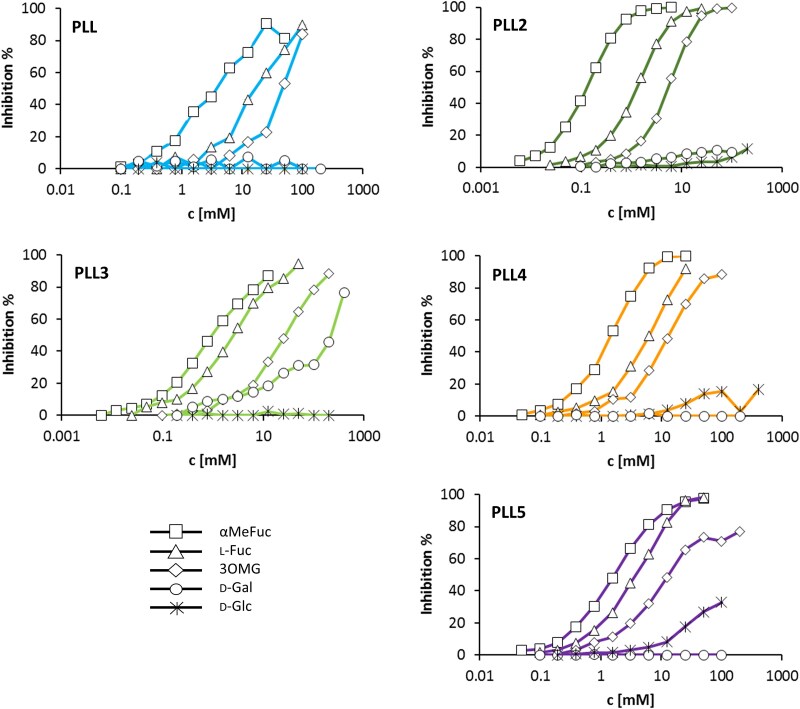
SPR inhibition assay. Comparison of the inhibitory effect of monosaccharides on lectin binding to the MGMR channel. Individual saccharides are coded in shape: Methyl-α-l-fucoside – Square, l-fucose – Triangle, d-galactose – Circle, d-glucose – Star, 3-*O*-methyl-d-glucose – Rhombus. Data for PLL2 and PLL3 were already published (Faltinek et al. 2019; Fujdiarová et al. 2021).

In SPR experiments with ligands (α-l-fucoside or MGMR) bound to the biosensor surface, apparent K_D_ (K_D_(app)) decreased to μM values for α-l-fucoside, and even up to nM values for MGMR (Table 1). This effect of involving multiple binding sites in binding an oligovalent binding partner—avidity—is also typical for lectins, and this mechanism compensates for the low affinity of individual interactions (Lee and Lee 2000). The lowest avidity effect can be seen for PLL3, the only monomeric protein in this family. The competitive inhibition with five selected saccharides was tested on the MGMR channel (Fig. 7). The determined IC_50_ values are given in Table 2. Interestingly, αMeFuc and l-fucose inhibited the interaction with *O*-methylated MGMR (with IC_50_ in the low mM range), showing that all ligands aim for the same binding site/sites. Different behaviour was seen for d-galactose, which inhibited only the binding of PLL3 and not PLL2, despite confirmed binding to both proteins from ITC, suggesting that d-galactose aims for different binding sites than MGMR in PLL2. Also, a clear difference between the inhibitory effect of 3-*O*-methyl-d-glucose (3OMG, IC_50_ in the low mM range) and d-glucose (no inhibition at the highest concentration tested, 200 mM) was shown, highlighting the importance of the 3-*O*-methyl group in binding affinity.

**Table 1:** SPR experiments.

	PLL	PLL2	PLL3	PLL4	PLL5
α-l-fucoside	8.5 ± 0.2 μM	117 ± 4.8 μM	322 ± 86 μM	4.3 ± 0.1 μM	64 ± 8.6 μM
MGMR	44 ± 4.6 μM	6.1 ± 0.2 nM	607 ± 46 μM	0.8 ± 0.06 μM	0.3 ± 0.03 μM

K_D_(app)s of PLL family lectins binding to immobilized α-l-fucoside or 3,6-*O*-Me_2_-d-Glcβ1-4(2,3-*O*-Me_2_)-l-Rhaα (MGMR). Standard deviations were calculated from three individual measurements. Data for PLL2 and PLL3 were already published (Faltinek et al. 2019; Fujdiarová et al. 2021).

**Table 2:** Inhibition tests using SPR.

IC_50_ [mM]					
inhibitor protein	d-Gal	αMeFuc	l-Fuc	d-Glc	3OMG
PLL	> 200	4.0 ± 1.35	19.5 ± 7.50	> 200	48.1 ± 14.88
PLL2	> 100	0.1 ± 0.01	1.2 ± 0.04	> 400	5.2 ± 0.91
PLL3	156 ± 53.9	1.0 ± 0.05	2.5 ± 0.09	> 400	26.4 ± 2.84
PLL4	> 200	1.4 ± 0.04	5.7 ± 0.21	> 400	13.1 ± 0.84
PLL5	> 200	1.7 ± 0.13	3.6 ± 0.13	> 200	16.9 ± 2.69

Inhibitory effect of selected saccharides on PLLs binding to immobilized 3,6-*O*-Me_2_-d-Glcβ1-4(2,3-*O*-Me_2_)-l-Rhaα (MGMR). IC_50_ was determined from a chart of inhibitor dilution series vs RU response. Data for PLL2 and PLL3 were already published (Faltinek et al. 2019; Fujdiarová et al. 2021).

### Structure of PLLs in a complex with methyl-α-l-fucoside

**Fig. 8:**
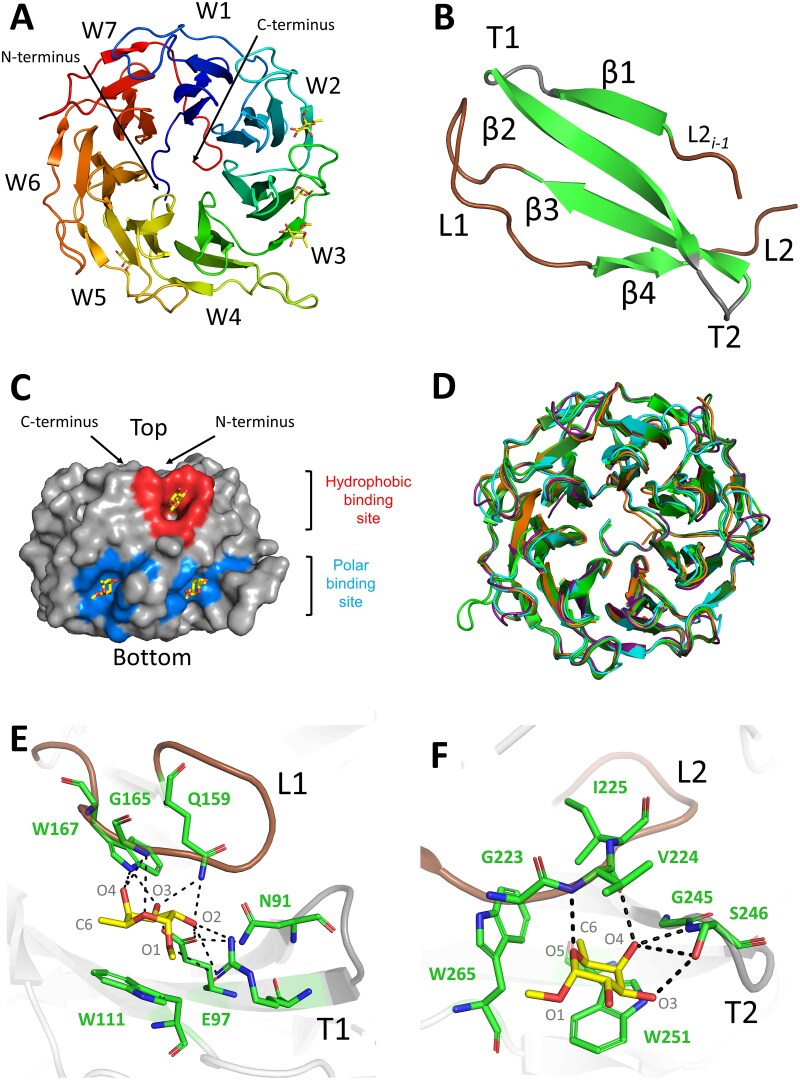
Structure of PLL3 in complex with αMeFuc. A) Seven-bladed β-propeller fold (cartoon; N to C rainbow), αMeFuc ligands are depicted as yellow sticks. Each blade is made of a W-motif labelled W1 – W7. B) a detailed look at the W-motif (cartoon) consisting of four β-strands (β1-β4) linked by two β-turns (T1 and T2) and two flexible loops (L1 and L2). C) Localisation of the polar and hydrophobic binding sites occupied with αMeFuc (yellow sticks), the example of PLL3. D) Structural alignment of all seven-bladed β-propeller lectins from *P. Laumondii* (PLL – Cyan, PLL2 – Dark green, PLL3 – Green, PLL4 – Orange, PLL5 – Violet). E) Polar type of site occupied by αMeFuc, an example of PLL3-2P site. Protein amino acids (green sticks) interact via polar contacts (dashed lines) with αMeFuc (yellow sticks). F) Hydrophobic type of site occupied by αMeFuc, an example of PLL3-4H site. Protein amino acids (green sticks) interact via polar contacts (dashed lines) with αMeFuc (yellow sticks).

The complexes of all studied lectins with αMeFuc were solved and deposited in the PDB database (PDB codes 8Q7U (PLL), 8Q80 (PLL2), 8Q81 (PLL3), 8Q82 (PLL4), and 8Q83 (PLL5)). All studied lectins belong to the seven-bladed β-propeller structural family (Fig. 8A). Each blade makes a W-motif composed of four antiparallel beta-strands (β1-β4) connected by β-turns and loops (Fig. 8B). The C and N termini are located on the same side of the torus (“top”) and create a cap of the inner cavity of the torus (Fig. 8C). The backbone RMSD between monomers of all studied lectins ranges from 0.449 Å to 0.696 Å where the main differences in the structures are flexible loops in the blades (Fig. 8D). The more variable section is the N-terminal part (approx. 30 amino acids) is not distinguishable in the crystal structure, except for the lectin PLL3, where the N-terminal methionine and a pair of connecting residues were localized in one of the potential binding sites (Faltinek et al. 2019). The function of the tail is not clear. It does not appear to contribute to the structural stability of the protein nor to ligand binding. They were not recognized as signal peptides by bioinformatical analysis, yet extracellular localization of PLLs is predicted by PSORTb v3.0, PSLpred, CELLO and SOSUI-GramN softwares (data not shown). Moreover, PLL2 and PLL3 were found by proteomic analysis in an extracellular fraction of *Photorhabdus* proteome (Turlin et al. 2006).

**Fig. 9:**
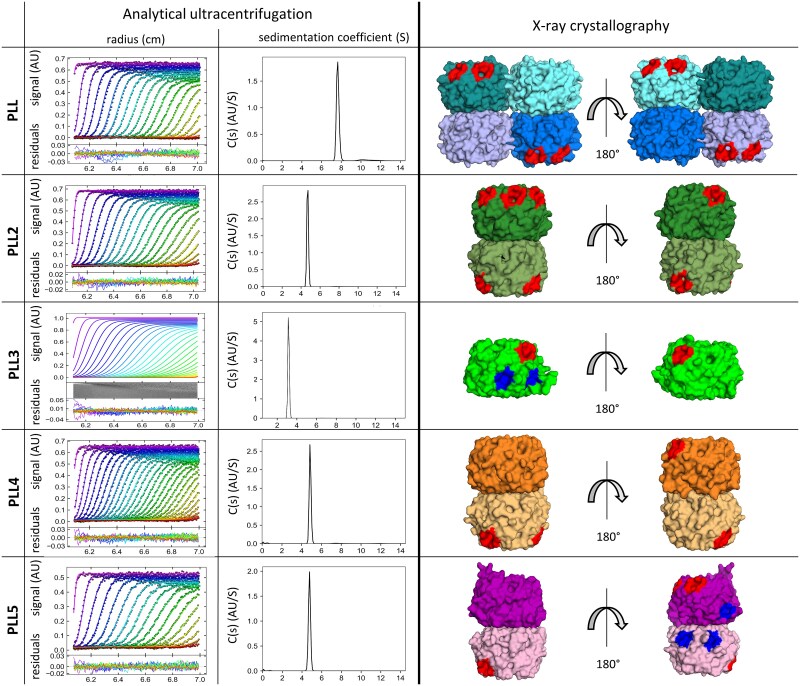
Oligomeric state of PLLs. Analytical ultracentrifugation: Sedimentation velocity profiles depicted. Every third recorded scan is shown (upper panel). The residual plots show the difference between experimental data and fitted curves (lower panel). Figures were created in GUSSI 1.4 (Brautigam 2015). Data for PLL2 and PLL3 are taken from (Faltinek et al. 2019; Fujdiarová et al. 2021). X-ray crystallography: Side view of PLLs. Individual monomers are color-coded. Binding sites occupied with αMeFuc are highlighted in blue (polar sites) or red (hydrophobic sites).

The oligomeric state in the X-ray structure differs for individual proteins across the family (Fig. 9). PLL3 is a monomer, as was previously described (Faltinek et al. 2019). PLL4 and PLL5 form homodimers in the same manner as PLL2 (Fujdiarová et al. 2021), where two monomeric units interact “bottom” to “bottom.” PLL is a homotetramer stabilized by up to four disulphide bridges. The electron density for the Cys267-Cys267 bridge shows one possible orientation of cysteines with sulphur atoms in 2.35 Å distance, corresponding to disulphide bond formation. Cys234 can adopt two different conformations, which can be explained as a mixture of oxidised and reduced states. Therefore Cys234-Cys234 bridge might not be formed in all PLL molecules (Fig. S7). Our data from mass spectrometry (intact mass analysis) suggest the formation of both disulphide bridges, as PLL behaved as a covalent tetramer during this experiment (Fig. S5 panel D). The formation of disulphide bridges is remarkable, as the protein was produced in the *E. coli* strain, which does not support a disulphide bridge formation; however, this feature was already reported for homologous dimeric lectin PHL (Jančaříková et al. 2017) and homologous PLL from *P. kayaii* (Kumar et al. 2016). Other studied proteins do not form S-S bridges, as they either lack cysteines in the sequence or the existing cysteine is inside the binding site cavity.

The dimerization interface in PLLs is mediated by loops in blades W1-W7. Interestingly, two different mutual orientations of monomers are observed in formed dimers. The PLL chain A blade W1 interacts with chain B blade W3, whereas in the case of PLL2, PLL4 and PLL5, chain A blade W1 interacts with chain B blade W5 (Fig. S8). The oligomeric state was also studied in the solution using the analytical ultracentrifugation (AUC), the sedimentation velocity method. The results confirm the crystallography findings (Fig. 9). The PLL is a tetramer in solution (sedimentation coefficient 7.6 S); PLL4 and PLL5 form dimers in solution (sedimentation coefficients: PLL4 = 4.9 S and PLL5 = 4.7 S).

The saccharide binding sites are located between the adjacent blades and are uniquely organized in two separate circles around the monomer. We previously showed that these two circles of binding sites have different structural features and ligand specificity and were therefore named as hydrophobic (H, close to the “top” of the monomer) and polar type of sites (P, closer to the “bottom” of the monomer) (Jančaříková et al. 2017; Fujdiarová et al. 2021). H sites consist of a hydrophobic pocket deepening into the protein surrounded by mainly polar residues. The P sites are formed by a different set of mainly polar amino acids without a hydrophobic pocket. The binding sites 1H and 1P are located between the blades W1 and W2, the sites 2H and 2P between blades W2 and W3, and so forth, with up to seven hydrophobic and seven polar binding sites existing per monomer. In beta-propeller lectins, multivalency is achieved by two strategies, either oligomerization as in the case of lectin RSL (Kostlánová et al. 2005) or multiple binding sites existing within one monomer as in the case of lectins AAL (Wimmerova et al. 2003), PVL (Cioci et al. 2006) and lectins from the PLL family (Kumar et al. 2016; Fujdiarová et al. 2021).

**Fig. 10:**
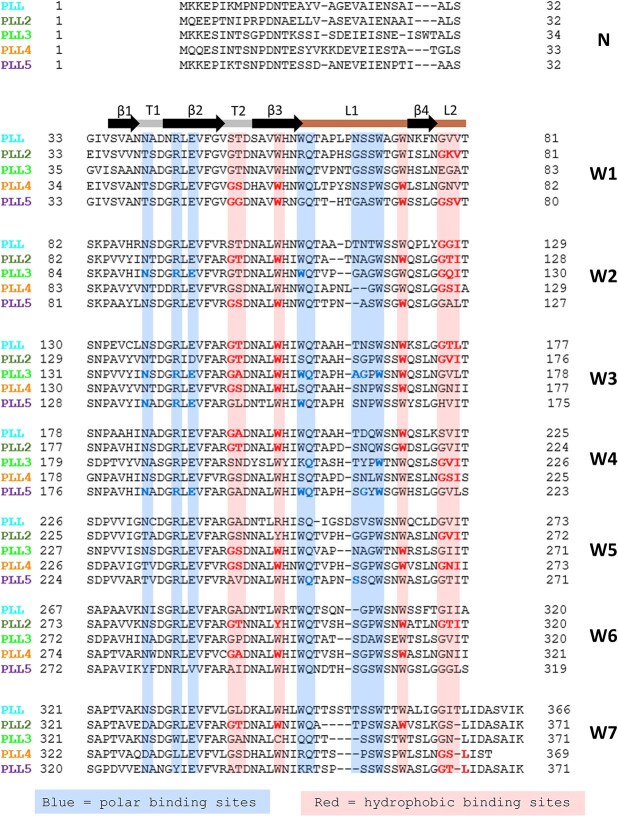
Sequence alignment of the PLL family lectins from *P. Laumondii.* Sequences used for analysis in Clustal omega (Sievers et al. 2014): PLL (NCBI reference sequence WP_011145107.1); PLL2 (NCBI reference sequence WP_011145109.1); PLL3 (NCBI reference sequence WP_011145110.1); PLL4 (NCBI reference sequence: WP_011145111.1); PLL5 (NCBI reference sequence: WP_011145112.1). Structural motives (W-motif, β-turns and loops) are schematically represented on the top. Amino acids interacting with αMeFuc in presented crystal structures are bold. Coloured backgrounds highlight amino acid conservation in polar and hydrophobic binding sites, respectively, across all members of the family.

The occupancy of the binding sites by αMeFuc is summarized in Table 3. αMeFuc occupied H sites in all PLLs but was found in P sites only in PLL3 and PLL5. The activity of both hydrophobic and polar binding sites has been confirmed crystallographically for the majority of PLLs (PHL (Jančaříková et al. 2017), PLL2 (Fujdiarová et al. 2021), PLL3, and PLL5: this study). For PLL and PLL4, only hydrophobic site activity has been shown, as αMeFuc occupied only H sites, and structure in complex with other potential ligands is not available. However, conserved ligand binding residues in most P sites in PLL and PLL4 (Fig. 10) presume their activity. Different ligand specificity of H and P sites have been demonstrated by crystal structures for lectins PLL2 (Fujdiarová et al. 2021) and PHL (Jančaříková et al. 2017). This has yet to be confirmed for the other members of the PLL family. Still, based on the high conservation of ligand binding sites within the family (Fig. 10), we hypothesize dual specificity is a feature of this lectin family. Dual specificity is not very common in lectins, but it was described in several cases, e.g. in two-domain lectin BC2L-C from *Burkholderia cenocepacia* (Šulák et al. 2011) or ABA lectin from *Agaricus bisporus* (Nakamura-Tsuruta et al. 2006). The existence of binding sites with different sugar specificity within one domain was described for LecA lectin from *Pseudomonas aeruginosa* (Blanchard et al. 2014). However, two sets of sites with different specificity within one lectin domain are unique and were not described outside the PLL lectin family.

**Table 3:** Site occupancy of PLLs/αMeFuc complexes.

		PLL	PLL2	PLL3	PLL4	PLL5
Hydrophobic sites	1H	^*^	ligand	^*^	^*^	ligand
	2H	ligand	ligand	ligand	ligand	
	3H	ligand	ligand	^*^	^*^	c
	4H	+	+	ligand	ligand	^*^
	5H		ligand	c	ligand	^*^
	6H	^*^	ligand	+	^*^	^*^
	7H	c	^*^	c	ligand	ligand
Polar sites	1P	^*^	+	c		+
	2P	^*^		ligand		
	3P	^*^	+	ligand	+	ligand
	4P		^*^	+	+	ligand
	5P	+		^*^		
	6P	+		c		^*^
	7P			+	+	+

Ligand – well defined electron density for ligand in binding site; ^*^– poorly defined electron density in binding site; c – binding site blocked by crystal contact; + − mutation of one of the residues involved in ligand binding.

Residues involved in ligand binding are highly conserved throughout the PLL family (Fig. 10). The orientation of the ligand in the H-type binding site is consistent across all PLLs (Fig. 8F - an example of PLL3-4H site). C6 of αMeFuc points inside the binding pocket, where it is stabilized via hydrophobic interaction. Two highly conserved tryptophans (e.g. W265 and W251 in PLL3-4H site) are essential in stabilizing the ligand via CH-π stacking interaction with C6, C5, and C4. Hydrogen bonds are mediated by the backbone of the β-turn T2 and the loop L2, stabilizing O4 and O5 of the αMeFuc molecule. Additional ligand stabilization is formed by a hydrogen bond between O3 and the side chain of the polar amino acid (Ser/Thr; e.g. S246 in PLL3-4H site) if presented in the β-turn T2. The methyl group on C1 of methyl-α-l-fucoside ligand is oriented towards the solvent, providing sufficient space to accommodate potential binding of methyl-β-l-fucoside in the H sites of PLLs. The ligand in the P-type binding site has a distinct orientation, where C6 is pointing outside the binding pocket (Fig. 8E - an example f PLL3-2P site). Delocalized electrons of the tryptophan (e.g. W111 in PLL3-2P site) are involved in the stabilization of C3, C4 and C5 via CH-π stacking interaction. Stabilization of the O1, O2, O3 and O5 in P-type binding sites is mainly mediated by hydrogen bonds of amino acid’s side chains, in contrast to H-type binding sites. The backbone of the loop L2 is involved in the stabilization of O4 via the hydrogen bond. In addition to αMeFuc ligand, electron density not assigned to a specific ligand was found in multiple binding sites. Crystal contacts block several binding sites, and several sites might not be active since some of the residues responsible for ligand binding are mutated.

## Conclusion

The current study brings new details of the biology of seven-bladed β-propeller lectins from *Photorhabdus laumondii* and their role in its complex life cycle. Based on the described PLLs behaviour, we suggest they participate in the interaction with the host and its immune system rather than in establishing a mutualistic relationship with nematode symbionts. PLLs showed interference with PO system and ROS production; they did not support the biofilm formation nor binding to a nematode host and preferred interaction with fucosylated saccharides, which are present on the effector proteins of the insect immune system. Future research, including the identification of natural ligands and binding specificity towards host immune cells, could help to understand why multiple lectins with similar structures are concurrently produced by bacteria *Photorhabdus* spp and their genes are closely coded in the bacterial genome. Our data from glycan array, ITC, and SPR suggest similar, but not identical, affinity of PLLs towards saccharides, and we also observed differences in their biological activity. The intriguing question for future studies is whether the PLL homologs can act in an orchestrated manner and provide spatial- or temporal-specific interactions between *Photorhabdus* and its host.

## Materials and methods

### Protein identification and cloning

PLL2 and PLL3 were identified and cloned as described previously (Faltinek et al. 2019, Fujdiarová et al. 2021). The hypothetical proteins PLL, PLL4 and PLL5 were identified with bioinformatic analyses of the genome of *Photorhabdus laumondii* subsp*. laumondii* TT01 (Taxonomy ID: 243265, NCBI reference sequence: BX470251.1) (Duchaud et al. 2003) using the NCBI BLAST tool (Altschul et al. 1990). The sequence of the native lectin PLL isolated from *Photorhabdus kayaii*, formerly *P. luminescens* subsp. *kayaii*, (Kumar et al. 2016) was used as a template for the analyses. NCBI reference sequences are: PLL - WP_011145107.1, PLL4 - WP_011145111.1, PLL5 - WP_011145112.1. Synthetic genes *pll, pll4* and *pll5* were prepared by Life Technologies (Thermo Fisher Scientific, Waltham, MA, USA) with the codons optimized for expression in *E. coli.* The restriction sites for NdeI and HindIII restriction endonucleases were added before and after gene sequences, respectively. The genes were separately cloned into the pET25b vector (Novagen, Darmstadt, Germany) using HindIII and NdeI restriction enzymes (NEB, Ipswich, MA, USA). The created vectors pET25b_*pll,* pET25b_*pll4,* and pET25b_*pll5* were transformed into *E. coli* DH5α and subsequently into the expression strain *E. coli* Tuner(DE3) (Novagen). The presence of the required vector in transformed cells was ensured by the presence of ampicillin resistance. The sequence of pET25b_ *pll,* pET25b_ *pll4,* and pET25b_ *pll5* was confirmed by sequencing of the re-isolated vector.

### Protein production and purification

PLL2 and PLL3 were produced and purified as described previously (Faltinek et al. 2019; Fujdiarová et al. 2021). Stock cells *E. coli* Tuner(DE3) pET25b_ *pll, E. coli* Tuner(DE3) pET25b_ *pll4* and *E. coli* Tuner(DE3) pET25b_ *pll5* were kept at −80 °C. Cells were inoculated in the low-salt LB broth with the addition of 100 μg/mL ampicillin and were cultivated at 37 °C to an OD_600_ of 0.5. Protein production was induced using 0.5 mM isopropyl-β-d-1-thiogalactoside and cells were cultivated for another 16 h at 18 °C. Cells were harvested by centrifugation at 12,000 g for 10 min. The cell pellet was resuspended in 20 mM Tris/HCl, 150 mM NaCl, pH 7.5 (Tris buffer) and stored at −20 °C until further use.

PLL, PLL4 and PLL5 were purified by affinity chromatography by the AKTA FPLC system (Cytiva, Marlborough, MA, USA). Cells were disrupted by sonication (VCX 500, Sonics & Materials, Inc., Newton, CT, USA). The cell lysate was separated by centrifugation at 21,000 g at 4 °C for 1 h and filtrated through a filter with a pore size of 0.22 μm (CarlRoth, Karlsruhe, Germany). The cell lysate was then loaded onto d-mannose-agarose resin (Sigma-Aldrich, St. Louis, Missouri, USA) equilibrated with the Tris buffer. PLL and PLL4 were eluted isocratically, and PLL5 was eluted by ultrapure water. The purity of eluted fractions was analysed using SDS-PAGE electrophoreses (12% gels stained with Coomassie Brilliant Blue R-250 or silver-stained, Supplementary Figs. S2-S5). The purified proteins were dialyzed against the buffer suitable for further studies.

### Glycan microarray

Lectins were fluorescently labelled with DyLight 488 according to the manufacturer’s instructions and the unbound dye was extensively dialyzed out of the protein solution. The microarray chip (Semiotik LLC, Moscow, Russia, batch OSPS280715) containing 634 saccharide ligands, each in 6 replicates, was treated with PBS (137 mM NaCl, 2.7 mM KCl, 8 mM Na_2_HPO_4_, 1.47 KH_2_PO_4_, pH 7.4) with added 0.1% Tween-20 for 15 min. The labelled proteins (200 μg/mL) were then separately applied to the chip surface and incubated in a humidity chamber at 37 °C for 1 h. After the incubation, the unbound protein was washed out with PBS containing 0.05% Tween-20 and deionized water. The chip was dried by airflow and scanned using an InnoScan1100 AL (Innopsys, Carbonne, France) with a 488 nm laser. The data were analyzed with the software Mapix 8.2.2 and an online glycan chip converter (Semiotic, https://rakitko.shinyapps.io/semiotik).

### Isothermal titration calorimetry (ITC)

ITC experiments were performed using an Auto-ITC200 calorimeter or Automated PEAQ-ITC calorimeter (both Malvern Panalytical, Malvern, UK). Measurements were carried out at 25 °C in the Tris buffer. Before the analyses, lectins were extensively dialyzed against the Tris buffer and the dialysate was used to dissolve ligands. The protein (0.1 mM or 0.2 mM) in the cell was titrated with 2 μL injections of the ligand (20 mM or 40 mM). Control titrations of buffer into buffer and ligand into buffer resulted in insignificant heat response. Experiments for each interacting ligand were carried out in triplicates. Integrated heat effects were evaluated by nonlinear regression using a single-site binding model in Origin 7 (OriginLab, Northampton, USA) (Wiseman et al. 1989). Triplicate measurements were fitted simultaneously using a global fit approach. Due to the low affinity of the interaction, the binding stoichiometry was fixed during the fitting.

### Analytical ultracentrifugation (AUC)

The oligomeric state of lectins in solution was investigated by AUC using a ProteomeLab XL-I analytical ultracentrifuge (Beckman Coulter, Indianapolis, IN, USA) equipped with an An-60 Ti rotor. Before analysis, proteins were dialyzed against the Tris buffer and the dialysate was used as an optical reference. Experiments were performed at various protein concentrations (0.03–0.25 mg/mL). Sedimentation velocity experiments were conducted in titanium double-sector centrepiece cells (Nanolytics Instruments, Potsdam, Germany) loaded with 380 μL of the protein and 380 μL of the reference solution. Data were collected using absorbance optics at 20 °C at a rotor speed of 48,000 or 50,000 rpm. Scans were performed at 280 nm and 0.003 cm spatial resolution in a continuous scan mode. The partial specific volume of the protein together with solvent density and viscosity were calculated from the amino acid sequence and the buffer composition, respectively, using the software Sednterp (http://www.jphilo.mailway.com/sednterp.htm). The sedimentation profiles were analyzed with the program Sedfit 16.1c (Schuck 2000). A continuous size distribution model for non-interacting discrete species was used to provide a distribution of sedimentation coefficients.

### Surface plasmon resonance (SPR)

SPR experiments were carried out on BIAcore T200 or BIAcore S200 instruments (GE Healthcare, UK). Sugar moieties were immobilized on the chip surface using the standard procedures according to the manufacturer’s instructions. Measurements with immobilized MGMR were performed on the chip prepared previously (high-density chip) (Fujdiarová et al. 2021). Measurements with immobilized α-l-fucose were performed on the streptavidin chip (GE Healthcare, UK). Biotinylated α-l-fucose was immobilized on channel 2 to yield a final response of 29 RU, unmodified channel 1 was used as a control.

Measurements were performed in Tris buffer with the addition of 0.05% Tween 20 at a flow rate of 30 μl/min at 25 °C. The protein contact time was 120 s for the determination of the apparent K_D_, or 60 s for the inhibition assays. 50 mM NaOH for 30 s (flow 30 μl/min) was used for chip regeneration. To determine K_D_s (app) towards MGMR, 2-fold dilution series of lectins was used (PLL: 0.048 – 50 μM, PLL4: 0.008 - 2 μM; PLL5: 1.9 – 500 nM). The data were evaluated with the BIAcore T200 or BIAcore S200 evaluation software using a steady state approach. To determine K_D_(app) towards α-l-fucose, 2-fold dilution series of lectins was used (PLL: 1.25 – 20 μM, PLL4: 0.39 – 12.5 μM; PLL5: 0.12 – 30 μM). The data were evaluated with the BIAcore T200 or BIAcore S200 evaluation software using a steady state approach (PLL5) or a single-cycle kinetics approach (PLL, PLL4). For SPR inhibition experiments on the MGMR channel, 250 nM PLL, 250 nM PLL4 or 62 nM PLL5, respectively, was used. Proteins of the given concentration were mixed with a concentration series of tested inhibitors (0.01 - 0.4 M). Proteins without an inhibitor were used as a control. The response of the lectin bound to the sugar surface at equilibrium was plotted against the concentration of inhibitor in order to determine IC_50_. The response in the appropriate blank channel was subtracted prior to all evaluations.

### Crystallization and data collection

The atomic structure of studied proteins in complex with αMeFuc was determined by protein crystallography. Lectins PLL2 and PLL3 were crystallized as previously described (Faltinek et al. 2019; Fujdiarová et al. 2021). The PLL2 crystals were transferred into the soaking solution (1 mM αMeFuc, 100 mM NaAc, pH 4.6, 10% PEG4000) for 120 min at 25 °C. The PLL3 crystals were transferred into the soaking solution (30 mM αMeFuc, 100 mM HEPES, pH 7.5, 10% PEG 8000) for 10 min at 25 °C. Crystals were cryoprotected in 40% PEG400 solution and then frozen in liquid nitrogen. PLL4 was concentrated to 10 mg/mL using an ultrafiltration cell with a 10 kDa cut-off membrane. The initial crystallization conditions were found using the commercial screening kits PACT, Classics, Classics Lite, and Classics II (Qiagen, Hilden, Germany). Commercial additive screens ANGSTROM (Molecular Dimensions, Sheffield, UK) and Hampton additive screen (Hampton Research, Aliso Viejo, CA, USA) were used to improve the diffraction properties of the PLL4 crystals. The final crystals of the PLL4/αMeFuc complex were obtained by co-crystallization of PLL4 with αMeFuc (25 mM final concentration in the drop) mixing in 1:2, 1:1, 2:1 ratio with precipitant solution: 20% PEG4000, 20% PEG300, 300 mM MgCl_2_, 100 mM MES pH 6.5. The PLL4 crystals were cryoprotected in 40% PEG400 solution and frozen in liquid nitrogen. Protein crystals of PLL and PLL5 were prepared by the batch method incubating PLL (8 mg/mL) or PLL5 (12 mg/mL) in 20 mM Tris/HCl, 150 mM NaCl, pH 7.5 solution, at 7 °C. The PLL crystals were transferred and incubated in a soaking solution (10 mM αMeFuc, 20 mM Tris/HCl, 150 mM NaCl, pH 7.5) for 6 min. The PLL5 crystals were transferred and incubated in a soaking solution (50 mM αMeFuc, 20 mM Tris/HCl, 150 mM NaCl, pH 7.5) for 5 min. The PLL crystals were cryoprotected in 40% PEG400 solution and the PLL5 crystals were cryoprotected in 50% erythritol solution and then frozen in liquid nitrogen. The diffraction data were collected at the PETRA III electron storage ring (DESY, Hamburg, Germany) or BESSY II electron storage ring (Berlin-Adlershof, Germany) (Table S2).

### Structure determination

Integration of the reflections from diffraction images was performed by XDS (Kabsch 2010) and XDSAPP (Krug et al. 2012). Data scaling and merging were performed by the Scala program from CCP4 v.8.0 program package (Winn et al. 2011) using 5% of data reserved for R_free_ calculation. Phase calculation was carried out by molecular replacement method using the program PHASER (PLL/αMeFuc, PLL3/αMeFuc, PLL4/αMeFuc) and the program Molrep (PLL2/αMeFuc, PLL5/αMeFuc). As an initial model for molecular replacement, PLL structure (PDB:5C9L) was used for complex PLL/αMeFuc and PLL5/αMeFuc; PLL2 structure (PDB:6RG2) was used for complexes PLL2/αMeFuc; and PLL3 structure (PDB:6 T96) was used for complex PLL3/αMeFuc, PLL4/αMefuc. Program COOT was used for model building and structure analyses (Emsley et al. 2010). All ligands were placed manually interpreting the Fo − Fc electron density map. Water molecules were placed by automatic search in the COOT and inspected manually. Refinement of the structures was carried out by Refmac5 (Murshudov et al. 2011). The final models were validated in PDBe validation server (https://pdbe.org) and deposited with PDB IDs: 8Q7U, 8Q80, 8Q81, 8Q82, and 8Q83. The refinement statistics are given in Table S2.

### Cultivation of *Photorhabdus laumondii* for gene expression analyses

Primary phase cells of *P. laumondii* subsp. *laumondii* TT01 (kindly provided by Dr. David Clarke, School of Microbiology and Microbiome Institute, University College Cork, Ireland) were inoculated from stock stored at −80 °C to 20 mL of LB and incubated in RTS-1C bioreactor (Biosan, Riga, Latvia) at 28 °C, 300 rpm, reverse spin period 60 s until a stationary culture was reached. The culture was then diluted to OD_600_ of 0.05, and the total volume of 20 mL was further incubated (28 °C; 300 rpm; reverse spin period 60 s) with measurement of the OD_850_ and growth rate every 10 min. The detection of optical density at a near-infrared wavelength of 850 nm using the bioreactor RTS-1C enables real-time, non-invasive monitoring of bacterial growth kinetics under aerobic conditions in Falcon tubes. The bacterial growth rate is determined from changes in suspension turbidity by the RTS software, which was calibrated for the size of *Photorhabdus* cells. Samples of the bacterial culture for gene expression analysis were collected at 4, 8, 24 and 48 hours after inoculation.

### Purification of total RNA and RT-qPCR analysis

Total RNA was extracted from 1 mL of the bacterial culture using the RNeasy^®^ Mini Kit (Qiagen, Hilden, Germany) including on-column DNase digestion according to the manufacturer’s protocol. The bacterial suspension was centrifuged (10,000 g; 2 min; RT), and the pellet was incubated for 10 min at 37 °C with TE buffer (Thermo Fisher Scientific, Waltham, MA, USA) containing lysozyme (1 mg/mL; Merck, Burlington, MA, USA) prior to the RNA extraction. The quantity and quality of total RNA were assessed using the spectrophotometer NanoDrop 1000 (Thermo Fisher Scientific, Waltham, MA, USA). The cDNA template for two-step RT-qPCR was synthesized from 1 μg of the extracted total RNA using the reverse transcriptase and other reagents purchased from Thermo Fisher Scientific (Waltham, MA, USA). The qPCR was carried out using LightCycler 480 (Roche, Basel, Switzerland) in 20 μL containing 1.5 μL of cDNA as a template, SYBR Green I Master kit (Merck, Burlington, MA, USA), and specific primers for the reference genes and genes coding for PLLs (Table S3). The amplification consisted of an initial denaturation for 5 min at 95 °C, followed by 45 cycles of 10 s denaturation at 95 °C, annealing for 10 s at 65 °C and extension for 10 s at 72 °C. Melting curves were analyzed for each reaction to ensure that each curve contained a single peak. PCR products were also run on agarose gels to confirm the presence of a single product with the expected length and confirmed by the sequencing. The relative expression of genes coding for PLLs was calculated using formula 2^–∆Ct^, in which ∆Ct is the difference of Ct values between the gene of interest and the average Ct value of four used housekeeping genes (16S rRNA, recA, gyrB, and UdP).

### Biofilm formation

The biofilm assay was performed according to a previous publication (Hapeshi et al. 2019). The liquid culture of *P. laumondii* in LB was diluted to OD_600_ of 0.05 and immediately used to inoculate a 24-well tissue culture plate (500 μL per well) containing 100 μg of tested lectin in 25 μL of Tris buffer or the same volume of the Tris buffer without protein as a control. Plates were incubated stationary for 24 h at 28 °C. After the incubation, the supernatant was discarded, and wells were washed three times with PBS. The plate was left to dry for one hour and incubated for 30 min with 0.2% crystal violet to stain adherent bacteria. After discarding the crystal violet solution, plates were washed three times with PBS, and any residual dye stuck in bacterial biofilm was released by a solution containing 70% ethanol and 10% isopropanol. The released dye was quantified by measuring absorbance at 570 nm using the plate reader Tecan Sunrise (Männedorf, Switzerland). We also prepared wells containing tested proteins and 500 μL of LB without bacteria to reveal a possible interference between tested lectins and the used staining. This control was further processed as described above.

### Interaction of PLLs with nematodes

The binding of PLLs to developmental stages of *Heterorhabditis bacteriophora* strain H221, (isolated from Pouzdřany, Czech Republic and kept in the laboratory collection at the Masaryk University, Brno), the nematode symbiont of *Photorhabdus,* was tested with lectins labelled with a green fluorophore DyLight™ 488 prepared as described above. Nematode eggs, larvae, and adults, including males, females and hermaphrodites, were collected from *Galleria mellonella* larvae that were reared on the artificial diet prepared according to Haydak (Haydak 1936) and kept at 29 °C in the constant dark. *G. mellonella* larvae are commonly used to culture *H. bacteriophora*. Infective juveniles were collected from White traps at least one week after they emerged from infected larvae. Each nematode stage was prepared in 20 μL of Tris buffer and incubated for 10 min with 20 μL of labelled lectin (1.3 – 1.6 mg/mL) or the same volume of Tris buffer as a negative control. To confirm the specificity of lectin binding, we used bovine serine albumin labelled with DyLight™ 488 (1.6 mg/mL) as a negative protein control, and to verify the binding ability of PLLs, we used fucosylated beads as a positive control. After incubation, the nematode stages were washed two times with PBS and observed using an SP8 laser scanning confocal microscope (Leica, Wetzlar, Germany).

To prepare nematode microsections, IJs collected from the White trap were washed in tap water filter-sterilized using 0.22 μm cellulose acetate membrane (Whatman, Maidstone, United Kingdom), embedded in Tissue-Tek^®^ O.C.T. Compound (Sakura Finetek, CA, USA), and frozen at −80 °C. Frozen blocks with nematodes were cut using cryostat Leica CM1850 to 12 μm thick sections. The sections were collected to SuperFrost™ Plus slides (Thermo Scientific, Waltham, MA, USA), fixed 10 min in 4% formaldehyde (pH 6.9; Merck, Darmstadt, Germany), and then washed 3 × 5 min in PBS (pH 7.4). Following wash, the sections were blocked in 1% BSA for one hour at room temperature and then treated with labelled lectins or labelled BSA (1.0 mg/mL) overnight in the dark at 4 °C. After incubation, the nematode sections were washed 3 × 5 min in PBS (pH 7.4), mounted in Vectashield^®^ HardSet™ antifade medium (Thermo Scientific, Waltham, MA, USA) and observed using an SP8 laser scanning confocal microscope (Leica, Wetzlar, Germany).

### Phenoloxidase (PO) activity in insect hemolymph

Basal phenoloxidase and total phenoloxidase activity were measured according to the previously published protocol (Fujdiarová et al. 2021) with minor modifications. Basal PO activity was measured in pooled hemolymph samples collected from the last instar of *G. mellonella* larvae and diluted 20x in ice-cold PBS (pH 7.0). Hemolymph samples were frozen at −80 °C until melted on ice, centrifuged (15 min; 12,000 g; 4 °C), and supernatants were further used for the measurements. Basal PO activity and total PO activity resulting from the treatment of hemolymph supernatant with α-chymotrypsin were both measured in the presence of tested lectin (100 μg in 25 μL of Tris buffer) or control (25 μL Tris buffer without lectin). PO activity was determined as the melanisation rate in 60 min of reaction measured at 492 nm using plate reader Sense (Hidex, Turku, Finland) and expressed as the integral of the reaction curve.

### Reactive oxygen species production in human blood

The effect of PLLs on the production of reactive oxygen species (ROS) was measured using the same protocol as published previously (Fujdiarová et al. 2021). Human blood type A samples were collected from healthy donors into tubes without anticoagulants and anonymized. All blood samples were used for ROS measurement within one hour. Briefly, the effect was measured using the dose of 100 μg of PLLs in 25 μL of Tris buffer and 2 μL of the whole human blood. Hank’s Balanced Salt Solution (HBSS; 0.137 M NaCl, 5.4 mM KCl, 0.44 mM KH_2_PO_4_, 0.25 mM Na_2_HPO_4_, 4.2 mM NaHCO_3_, 1.0 mM MgSO_4_, 1.3 mM CaCl_2_, 5.55 mM glucose, pH 7.4) and 10 mM luminol were used as the assay buffer and luminophore, respectively. Luminescence was measured in counts per second (CPS) for 120 min at 37 °C using the luminometer Chameleon V (Hidex; Turku; Finland). ROS production was expressed as the integral of the luminescence curve. We measured the constitutive ROS production in non-immune-activated human blood, and after activation with zymosan A from *Saccharomyces cerevisiae* (2.5 mg/mL in HBSS), N-Formyl-Met-Leu-Phe (fMLF; 10 μg/mL in HBSS), and phorbol 12-myristate 13-acetate (PMA; 10 μg/mL in HBSS).

### Statistical analysis

Data were statistically analysed in Prism 9 (GraphPad Software, San Diego, CA, USA). All data were assessed for normality and homogeneity of variance before further statistical analysis. If all presumptions were fulfilled, one-way ANOVA with post hoc Tukey’s HSD test was used to compare the tested groups; otherwise, a nonparametric Kruskal-Wallis test with post hoc Dunn’s test was used. Unpaired t test was used to compare the mean between two independent groups. Results with p-values less than 0.05 were considered statistically significant.

## Supplementary Material

Supporting_Information_S1_cwaf033

Table_S4_Glycan_array_raw_data

## Data Availability

Lectin structures are deposited in the PDB database and are publicly available with PDB IDs: 8Q7U, 8Q80, 8Q81, 8Q82, and 8Q83. All other experimental data can be available upon request; please contact Michaela Wimmerová, michaw@chemi.muni.cz.
