# Supplementary material for: The insight into the biology of five homologous lectins produced by the entomopathogenic bacterium and nematode symbiont Photorhabdus laumondii
Source: Glycobiology. 2025 Jun 3;35(7):cwaf033. doi: 10.1093/glycob/cwaf033 (PMC12203525; doi:10.1093/glycob/cwaf033)
Supplement: Supporting_Information_S1_cwaf033 [file supporting_information_s1_cwaf033.docx]

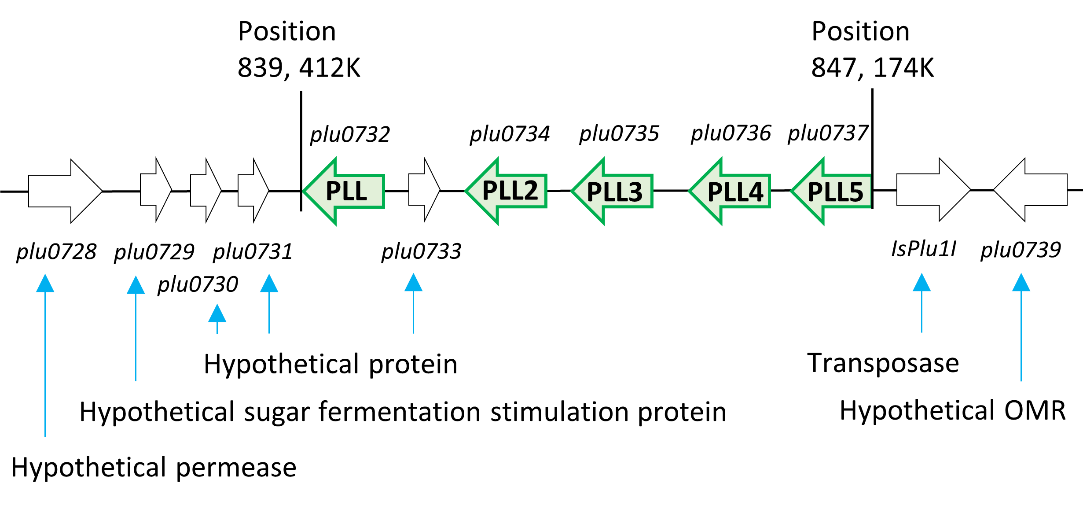


Figure S1: Schematic representation showing the localization of PLL-coding genes and their surroundings in the *Photorhabdus* genome. Whole genome of *P. laumondii* subsp *laumondii* TT01 (NCBI identifier BX470251.1) was used as a template. OMR = outer membrane receptor.


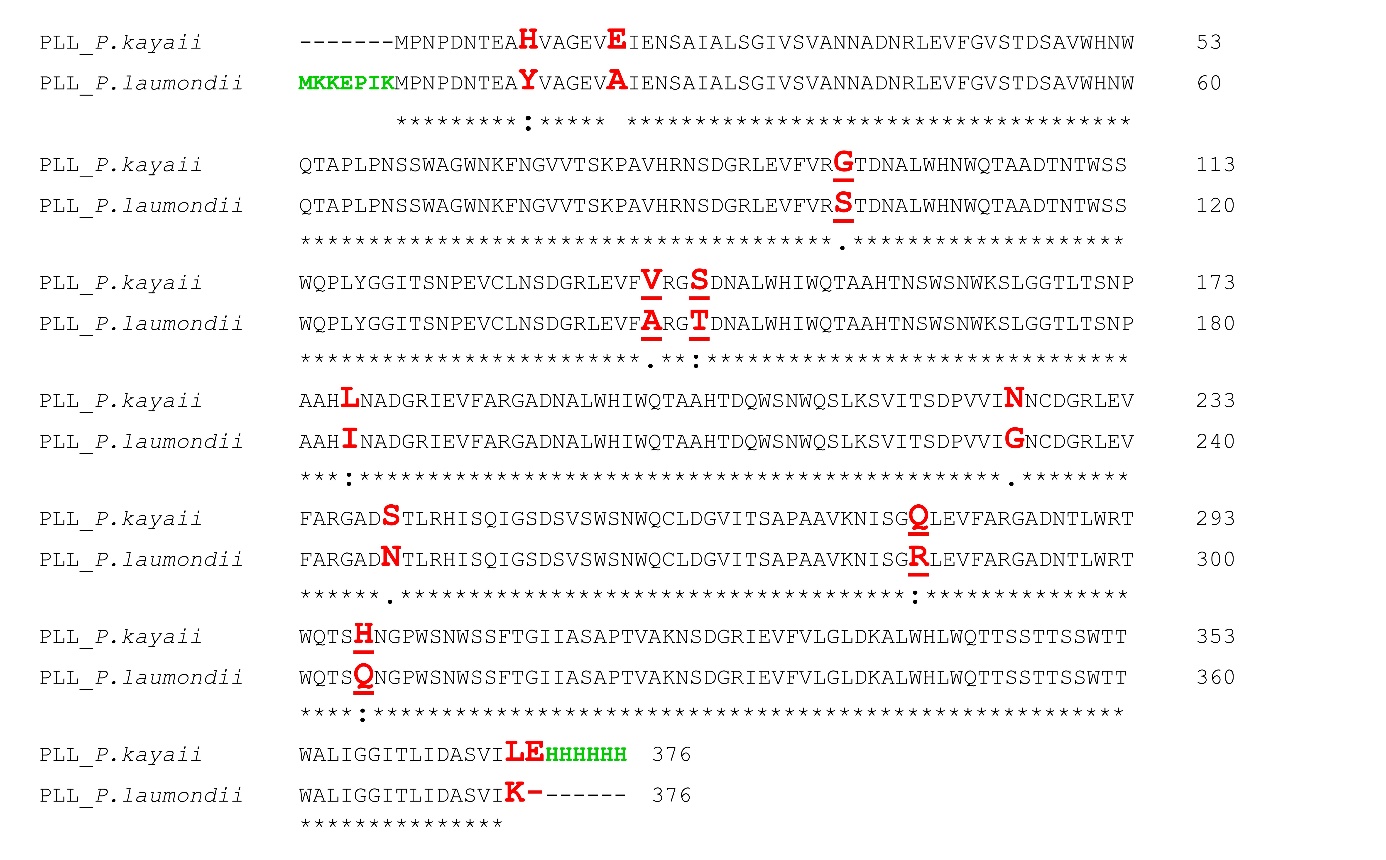


Figure S2: Differences in the PLL lectin sequences of *P. kayaii* and *P. laumondii*. Mutated amino acids are highlighted in red, and those involved in binding pocket formation are underscored. C-term His tag and 7 extra N-term residues are highlighted in green.


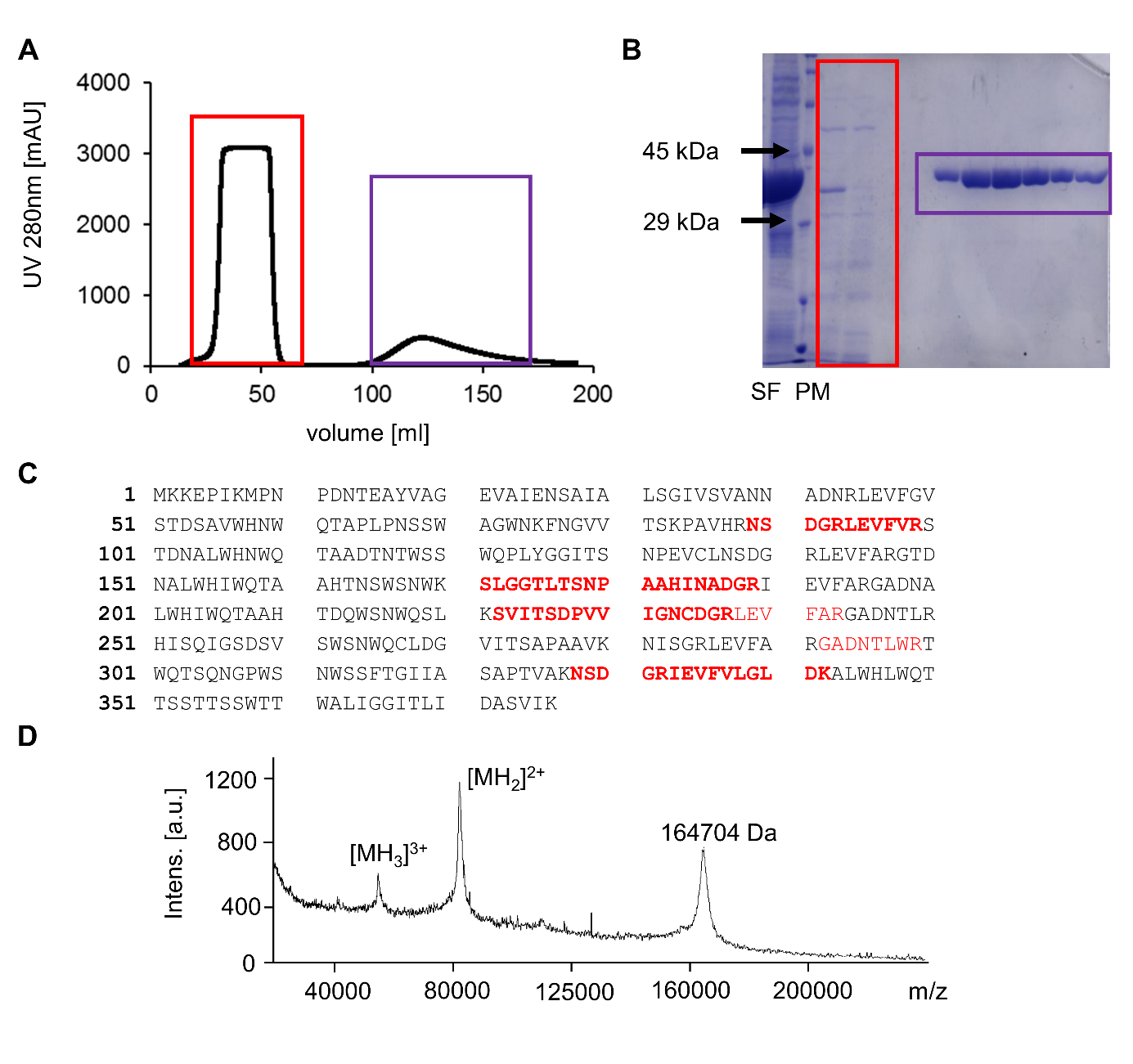


Figure S3**.** PLL purification and identity. (A) Representative record of PLL purification using affinity chromatography on d-mannose-agarose resin and (B) corresponding SDS-PAGE gel. SF – soluble fractions, PM – Protein marker III (AppliChem). Unbound fractions are highlighted by the red rectangle, fractions with PLL used for further analysis are highlighted by the violet rectangle. (C) Sequence coverage of PLL from MALDI MS/MS analysis. PLL sample was digested by trypsin for 2 hours at 40°C and subsequently analysed by MALDI MS/MS. The confirmed sequence is in bold and red, uncertain regions are red, undetected parts are black. (D) PLL intact mass analysis showed molecular mass 164,704 Da, which corresponds to the protein tetramer with full-length sequence including initial methionine. MALDI MS/MS and intact mass analyses we performed at a Proteomics Core Facility, CEITEC MU, Brno.


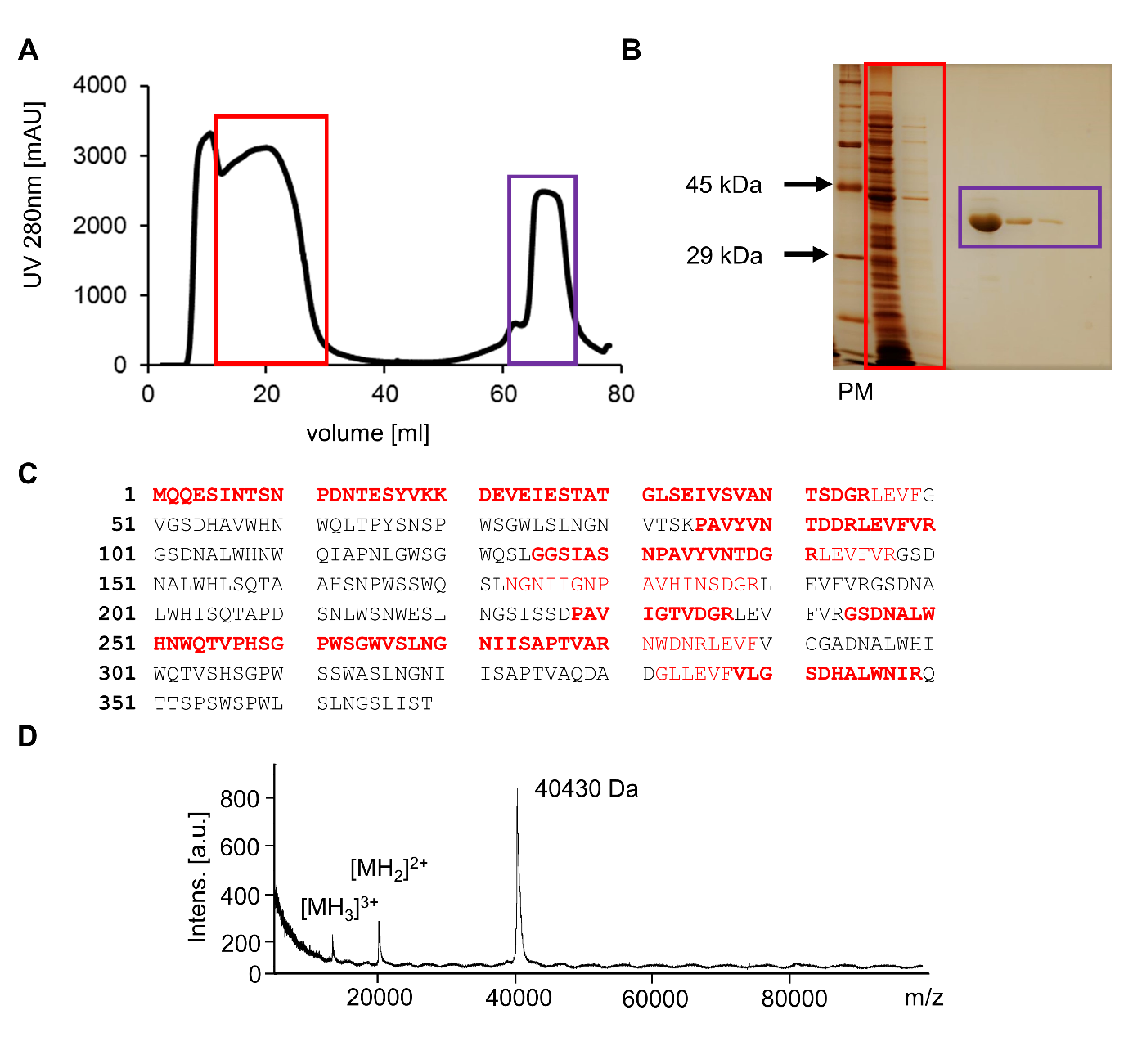


Figure S4**.** PLL4 purification and identity. (A) Representative record of PLL4 purification using affinity chromatography on d-mannose-agarose resin and (B) corresponding SDS-PAGE gel. PM – Protein marker III (AppliChem). Unbound fractions are highlighted by the red rectangle, fractions with PLL4 used for further analysis are highlighted by the violet rectangle. (C) Sequence coverage of PLL4 from MALDI MS/MS analysis. PLL4 sample was digested by trypsin for 2 hours at 40°C and subsequently analysed by MALDI MS/MS. The confirmed sequence is in bold and red, uncertain regions are red, and undetected parts are black. (D) PLL4 intact mass analysis showed molecular mass 40 430 Da, which corresponds to the full-length sequence including initial methionine. MALDI MS/MS and intact mass analyses we performed at a Proteomics Core Facility, CEITEC MU, Brno.


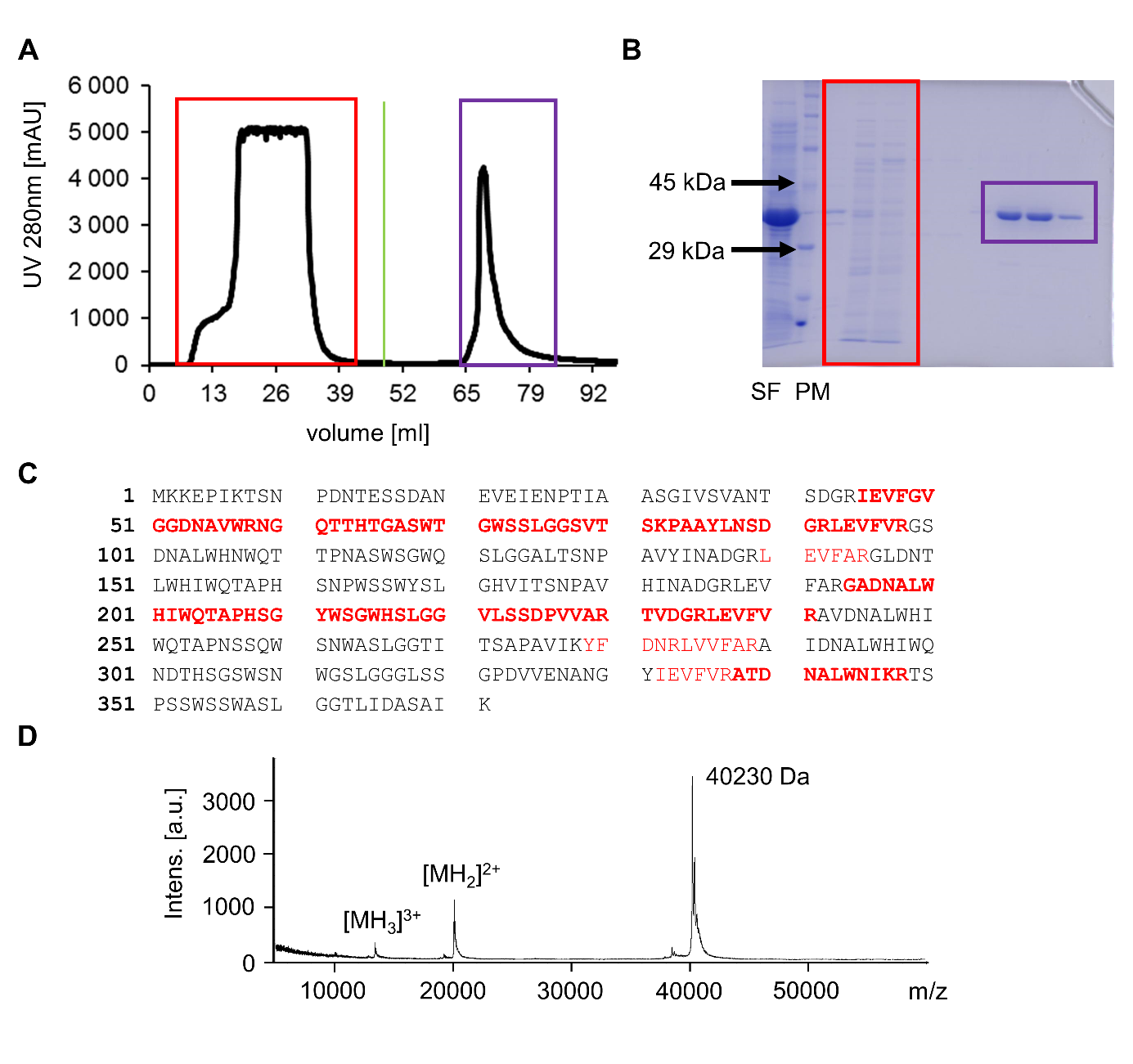


Figure S5**.** PLL5 purification and identity. (A) Representative record of PLL5 purification using affinity chromatography on d-mannose-agarose resin and (B) corresponding SDS-PAGE gel. SF – soluble fractions, PM – Protein marker III (AppliChem). Unbound fractions are highlighted by the red rectangle, fractions with PLL5 used for further analysis are highlighted by the violet rectangle, the green line represents the loading of elution buffer. (C) Sequence coverage of PLL5 from MALDI MS/MS analysis. PLL5 sample was digested by trypsin for 2 hours at 40°C and subsequently analysed by MALDI MS/MS. The confirmed sequence is in bold and red, uncertain regions are red, undetected parts are black. (D) PLL5 intact mass analysis showed molecular mass 40 230 Da, which corresponds to the full-length sequence including initial methionine. MALDI MS/MS and intact mass analyses we performed at a Proteomics Core Facility, CEITEC MU, Brno.


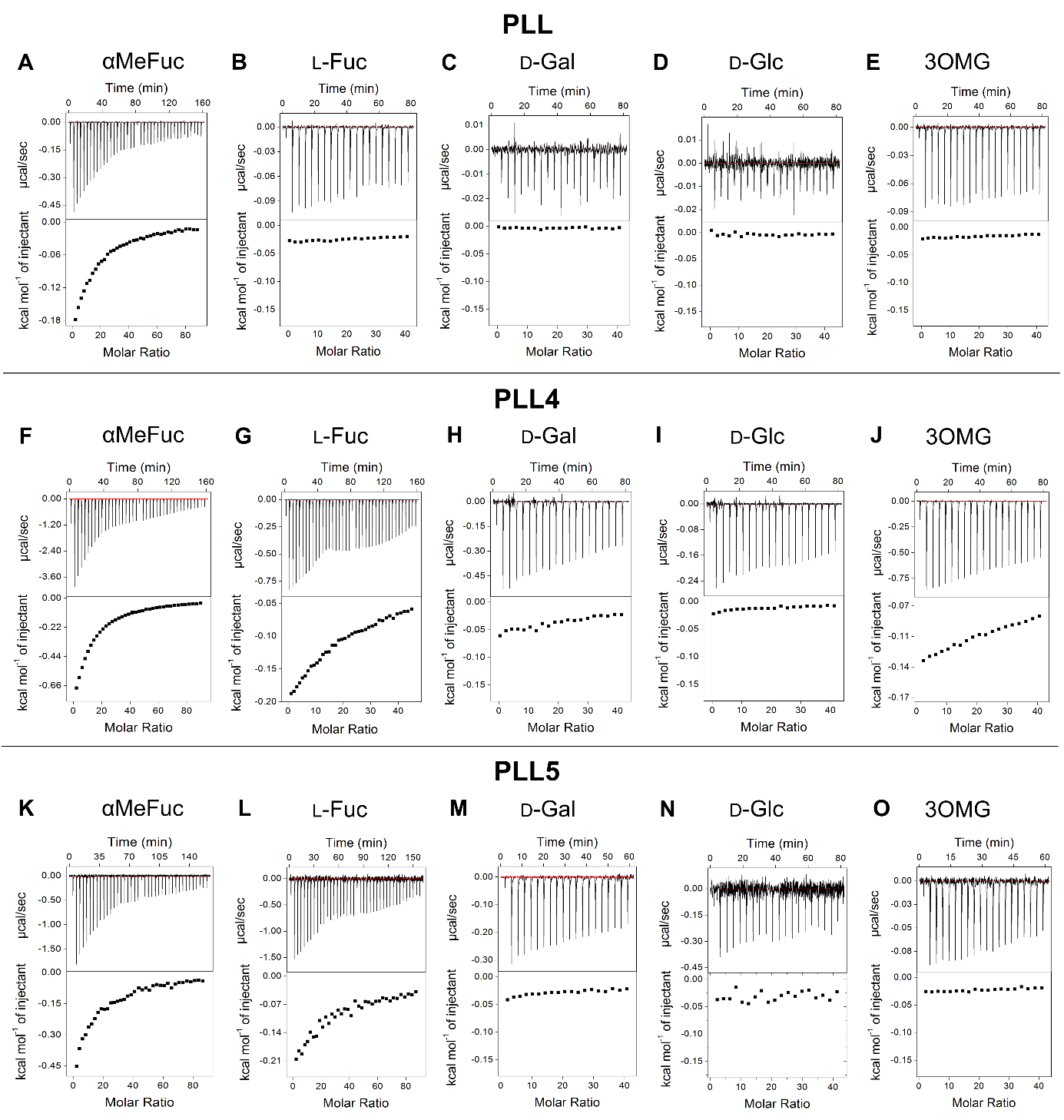


Figure S6: Representative ITC curves. Lectins PLL, PLL4 and PLL5 (100 µM or 200 µM) were titrated with monosaccharide ligands (20 mM or 40 mM). The baseline is highlighted in red.


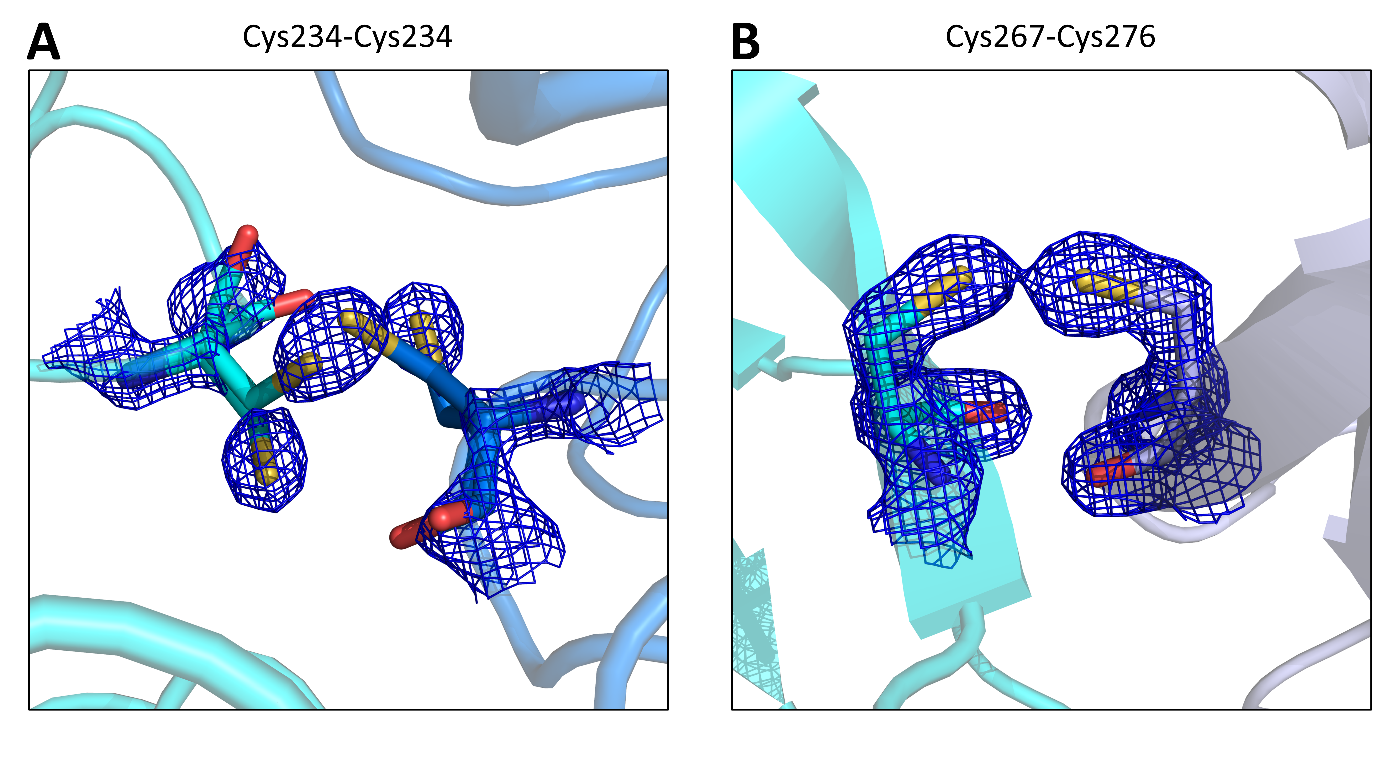


Figure S7: Disulphide bridges formed by PLL lectin (PDB: 8Q7U). Fobs-Fcalc electron density around ligands is displayed at 2.5σ as dark blue mesh and superposed with final cysteine positions after refinement. Cysteines are depicted as sticks, sulphur atoms are yellow.


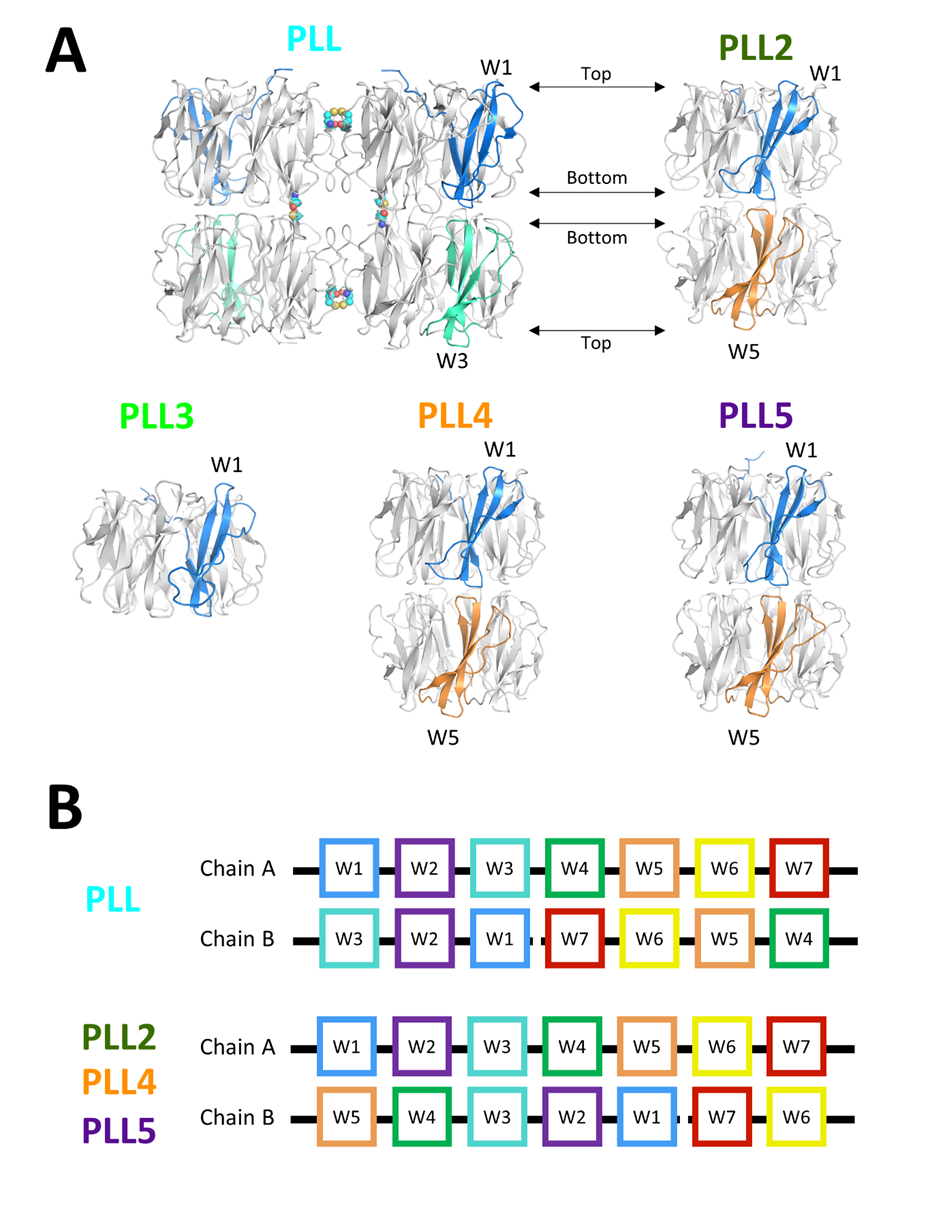


Figure S8: Oligomeric state of the seven-bladed β-propeller lectins from *P. laumondii.* (A) PLL (PDB: 8Q7U) forms a homotetramer, stabilized by four disulphide bridges (shown as spheres). PLL2 (PDB: 8Q80), PLL4 (PDB: 8Q82) and PLL5 (PDB: 8Q83) are homodimers. PLL3 (PDB: 8Q81) is a monomer in the crystal structure. The interaction of the monomers in a “bottom-to-bottom” manner is mediated by the β-turns T1 and the loops L1 of the blades W1-W7. (B) Schematic representation of opposing blades interaction in respect to Chain A to Chain B.

Table S1. **ITC experiments**. Dissociation constants of PLL family lectins titrated with selected monosaccharides. Stoichiometry (N) was fixed during the fitting procedure due to the low affinity. Standard deviations were calculated from three independent measurements. Stoichiometry was fixed to a value known from the X-ray structure when available. No int = no interaction. Data for PLL2 and PLL3 were already published ( Fujdiarová et al, 2021, Faltinek et al, 2019).

| K_D_ [mM] | N | αMeFuc | l-Fuc | d-Glc | 3OMG | d-Gal |
| --- | --- | --- | --- | --- | --- | --- |
| PLL | 2 | 3.6 ± 0.07 | No int | No int | No int | No int |
|  | 4 |  |  |  |  |  |
|  | 6 |  |  |  |  |  |
|  | 8 |  |  |  |  |  |
| PLL2 | 2 |  | 2.8 ± 0.07 |  |  | 0.5 ± 0.02 |
|  | 4 | 0.4 ± 0.02 | 2.6 ± 0.08 | 3.6 ± 0.21 |  | 0.4 ± 0.03 |
|  | 6 |  | 2.4 ± 0.08 |  |  | 0.3 ± 0.04 |
|  | 8 |  | 2.1 ± 0.10 |  | 11.2 ± 0.8 | 0.3 ± 0.06 |
| PLL3 | 2 |  | 3.6 ± 0.14 | 4.0 ± 0.12 | 11.5 ± 1.55 | 3.0 ± 0.06 |
|  | 4 | 0.7 ± 0.03 | 3.3 ± 0.12 | 3.6 ± 0.08 | 11.1 ± 1.50 | 2.7 ± 0.14 |
|  | 6 |  | 3.1 ± 0.10 | 3.3 ± 0.07 | 10.7 ± 1.50 | 2.5 ± 0.13 |
|  | 8 |  | 2.9 ± 0.10 | 3.1 ± 0.11 | 10.2 ± 0.62 | 2.2 ± 0.06 |
| PLL4 | 2 |  | 31.2 ± 0.95 | No int | No int | No int |
|  | 4 | 4.9 ± 0.08 | 29.9 ± 0.90 |  |  |  |
|  | 6 |  | 28.6 ± 0.89 |  |  |  |
|  | 8 |  | 27.2 ± 0.86 |  |  |  |
| PLL5 | 2 |  | 16.6 ± 1.02 | No int | No int | No int |
|  | 4 | 4.2 ± 0.17 | 16.0 ± 1.01 |  |  |  |
|  | 6 |  | 15.4 ± 1.00 |  |  |  |
|  | 8 |  | 14.7 ± 0.99 |  |  |  |

Table S2: Data collection and refinement statistics. Values in parentheses correspond to the highest resolution shell.

|  | PLL/αMeFuc (PDB:8Q7U) | PLL2/αMeFuc (PDB:8Q80) | PLL3/αMeFuc (PDB: 8Q81) | PLL4/αMeFuc (PDB: 8Q82) | PLL5/αMeFuc (PDB: 8Q83) |
| --- | --- | --- | --- | --- | --- |
| Beam line | PETRA, P14 | PETRA, P13 | PETRA, P13 | PETRA, P14 | BESSY, 14.1 |
| WaveLength (Å) | 0.9763 | 0.9800 | 0.9763 | 0.9763 | 0.9184 |
| SpaceGroup | I222 | P21 | P212121 | P21 | P21 |
| Unit-cell parameters  a/b/c (Å)  α/β/γ (°) | 71.22/89.18/158.77  90/90/90 | 68.16/85.68/68.19  90/102.06/90 | 56.61/69.62/76.25  90/90/90 | 70.01/82.86/70.30  90/105.36/90 | 93.71/102.94/97.48  90/90.03/90 |
| Resolution range (Å) | 45.57 – 1.60  (1.69 – 1.60) | 85.68 – 1.85  (1.95 – 1.85) | 76.25 – 1.50  (1.58 – 1.50) | 46.28 – 1.95  (2.06 – 1.95) | 45.52 – 1.70 (1.79 – 1.70) |
| Total reflections measured | 904519  (125033) | 249816  (36756) | 641273  (90549) | 388897 (53437) | 767548 (105199) |
| Unique reflections | 66905 (9679) | 64783 (9343) | 48191 (6850) | 56507 (8229) | 200282 (28794) |
| I/Sig (I) | 18.6 (2.8) | 11.4 (2.3) | 15.4 (3.1) | 21.7 (6.8) | 6.2 (1.6) |
| CC1/2 (%) | 99.9 (82.8) | 99.9 (78.5) | 99.9 (89.6) | 99.9 (97.8) | 99.3 (46.6) |
| Completeness (%) | 100 (100) | 99.1 (98.5) | 98.5 (97.2) | 100.0 (100.0) | 98.8 (97.8) |
| R merge | 0.084 (1.003) | 0.057 (0.630) | 0.097 (0.798) | 0.045 (0.231) | 0.118 (0.784) |
| Multiplicity | 13.5 (12.9) | 3.9 (3.9) | 13.3 (13.2) | 6.9 (6.5) | 3.8 (3.7) |
| Wilson B-factor | 18.8 | 31.0 | 15.1 | 32.0 | 20.0 |
| R_work_ | 0.176 | 0.206 | 0.162 | 0.212 | 0.197 |
| R_free_ | 0.192 | 0.235 | 0.186 | 0.246 | 0.213 |
| Reflection used for R_free_ | 3371 | 3207 | 2382 | 2799 | 10535 |
| RMSD bond  lengths | 0.0112 | 0.0070 | 0.0093 | 0.0077 | 0.0040 |
| RMSD bond angles | 1.4788 | 1.3048 | 1.4955 | 1.4327 | 1.2617 |
| Average B-factor | 21.0 | 35.0 | 17.0 | 35.0 | 21.0 |
| No. waters | 316 | 144 | 221 | 162 | 650 |
| No. of non-H atoms | 3185 | 5496 | 2960 | 5459 | 11424 |
| Ramachandran outliers | 0 | 2 | 0 | 0 | 0 |

Table S3: Primer sequences and the corresponding target genes, including housekeeping genes (HKG), used for RT-qPCR.

| Target gene | Amplicon size (bp) | Sequence of forward primer 5´-3´ | Sequence of reverse primer 5´-3´ | Reference |
| --- | --- | --- | --- | --- |
| 16S rRNA (HKG) | 305 | ACAGAGTTGGATCTTGACGTTACCC | AATCTTGTTTGCTCCCCACGCTT | Daborn et al, 2001 |
| Recombinase A (recA; HKG) | 233 | GTTCAATGGACGTTGAAACTATCTC | ATCAACACCCAACTTCTTAGCATAG | Mouammine et al, 2017 |
| DNA gyrase subunit B (gyrB; HKG) | 250 | ATACACGAAGAAGAAGGTGTTTCAG | TACCTGTCTGTTCAGTTTCTCCAAC | Mouammine et al, 2017 |
| Uridine phosphorylase (UdP; HKG) | 142 | TAACGGCCTCTTCCGATACT | AAAGTTGCCGATTCCATTTC | Krin et al, 2008 |
| PLL | 222 | CGCCGCTGATACCAATACCT | TTGATATGTGCAGCCGGGTT | This study |
| PLL2 | 297 | AAGCGTCGATTAAGCTGCCT | GCGTGATTACCAGTGCTCCT | This study |
| PLL3 | 292 | AACCTGCATTAGGAGCGACC | GGTCCGTGGTCTAATTGGCA | This study |
| PLL4 | 79 | CAGCCTGACCAACCGAGATT | TGGAAGTATTCGTCCGTGGC | This study |
| PLL5 | 85 | ATGTGTCGTTTGCCCATTGC | AGTGGCGAACACCTCTGATG | This study |

References:

Daborn PJ, Waterfield N, Blight MA, Ffrench-Constant RH. 2001. Measuring Virulence Factor Expression by the Pathogenic Bacterium *Photorhabdus luminescens* in Culture and during Insect Infection. J Bacteriol. 183:5834–5839.

Faltinek, L., Fujdiarová, E., Melicher, F., Houser, J., Kašáková, M., Kondakov, N., Kononov, L., Parkan, K., Vidal, S., and Wimmerová, M. 2019. Lectin PLL3, a Novel Monomeric Member of the Seven-Bladed β-Propeller Lectin Family. *Molecules*. 24: 4540

Fujdiarová, E., Houser, J., Dobeš, P., Paulíková, G., Kondakov, N., Kononov, L., Hyršl, P., and Wimmerová, M. 2021. Heptabladed β‐propeller lectins PLL2 and PHL from *Photorhabdus* spp. recognize *O*‐methylated sugars and influence the host immune system. *FEBS J.* 288:1343–1365

Krin E, Derzelle S, Bedard K, Adib-Conquy M, Turlin E, Lenormand P, et al. 2008. Regulatory role of UvrY in adaptation of *Photorhabdus luminescens* growth inside the insect. Environ Microbiol. 10: 1118–1134.

Mouammine A, Pages S, Lanois A, Gaudriault S, Jubelin G, Bonabaud M, et al. 2017. An antimicrobial peptide-resistant minor subpopulation of *Photorhabdus luminescens* is responsible for virulence. Sci Rep. 7: 43670.
